# The role of tropical volcanic eruptions in exacerbating Indian droughts

**DOI:** 10.1038/s41598-021-81566-0

**Published:** 2021-02-01

**Authors:** Suvarna Fadnavis, Rolf Müller, Tanusri Chakraborty, T. P. Sabin, Anton Laakso, Alexandru Rap, Sabine Griessbach, Jean-Paul Vernier, Simone Tilmes

**Affiliations:** 1 Indian Institute of Tropical Meteorology, MoES, Pune, India; 2 Forschungszentrum Jülich GmbH, IEK7, Jülich, Germany; 3 Finnish Meteorological Institute, Kuopio, Finland; 4 School of Earth and Environment, University of Leeds, Leeds, UK; 5 Forschungszentrum Jülich GmbH, Jülich Supercomputing Center, Jülich, Germany; 6 National Institute of Aerospace, Hampton, VA USA; 7 NASA Langley Research Center, Hampton, VA USA; 8 National Center for Atmospheric Research, Boulder, USA

**Keywords:** Climate sciences, Environmental sciences, Chemistry, Physics

## Abstract

The Indian summer monsoon rainfall (ISMR) is vital for the livelihood of millions of people in the Indian region; droughts caused by monsoon failures often resulted in famines. Large volcanic eruptions have been linked with reductions in ISMR, but the responsible mechanisms remain unclear. Here, using 145-year (1871–2016) records of volcanic eruptions and ISMR, we show that ISMR deficits prevail for two years after moderate and large (VEI > 3) tropical volcanic eruptions; this is not the case for extra-tropical eruptions. Moreover, tropical volcanic eruptions strengthen El Niño and weaken La Niña conditions, further enhancing Indian droughts. Using climate-model simulations of the 2011 Nabro volcanic eruption, we show that eruption induced an El Niño like warming in the central Pacific for two consecutive years due to Kelvin wave dissipation triggered by the eruption. This El Niño like warming in the central Pacific led to a precipitation reduction in the Indian region. In addition, solar dimming caused by the volcanic plume in 2011 reduced Indian rainfall.

## Introduction

Droughts associated with weak South Asian summer monsoons have a very strong impact on regional water security with substantial socio-economic consequences affecting millions of people in the region^1,2^. Famines caused by droughts in the Indian region have historically caused the death of millions of people^3^. Several factors contribute to a weakening of the monsoon and the droughts associated with it; in particular El Niño^4^, regional land-use changes^5^, and anthropogenic aerosol forcing^6,7^.

The global monsoon precipitation responds to large volcanic eruptions^8,9^. For example, northern hemisphere (NH) monsoon precipitation is weakened by NH and equatorial volcanic eruptions, but is enhanced by Southern hemisphere (SH) eruptions^10^. Droughts in West Africa also show linkages with asymmetric hemispheric volcanic forcing, i.e. volcanic eruptions in the NH produce droughts, whereas those in the SH induce a greening of the Sahel^11^. Furthermore, precipitation reductions during the years after tropical volcanic eruptions, mostly in the monsoon regions, have been found for five explosive eruptions in Coupled Model Intercomparison Project (CMIP5) models (Krakatau, Santa María, Agung, El Chichón, and Pinatubo) and in an observational analysis^12^. A decrease in precipitation following large volcanic eruptions is also consistent with an observed reduction in river streamflow in wet tropical regions^13,14^. Similar mechanisms were also found in simulations of volcanic forcing during the past two centuries, namely precipitation decreases in the tropics and subtropics, a weaker monsoon in the year after large eruptions^15^ and a shift of the intertropical convergence zone and the associated precipitation away from the hemisphere with greater volcanic forcing^16^. Consistently, climate proxy data over the past millennium derived from tree rings, ice cores, and speleothems show that volcanic forcing may drive a weak Asian summer monsoon in the second year after an eruption^17,18^.

Solar geoengineering studies investigating continuous injections of sulphur into the stratosphere assume an enhancement of the stratospheric aerosol burden resembling that caused by volcanic eruptions. Geoengineering studies consistently find a weakening of the Asian summer monsoon and a reduction of the associated precipitation^19,20^. Recent studies^21,22^, using four injection locations to minimise global, pole-to-equator and interhemispheric surface temperature gradient changes, found that while global annual precipitation over land is not affected, the heating of the lower tropical stratosphere results in important regional changes (e.g. a weakening of the Asian summer monsoon). While the impact of geoengineering is likely different to that of volcanic eruptions because of the assumed continuous application of geoengineering and the possibility to modulate injection locations to prevent precipitation shifts^23^, common processes impacting the Asian summer monsoon may be applicable to both.

The impact of moderate volcanic eruptions on tropical climate and the mechanisms of precipitation changes in response to such eruptions (e.g. changes in the South Asian summer monsoon) have hitherto received little attention. Here we employ 145-year long observational records (1871–2016) of volcanic eruptions and monsoon precipitation to investigate Indian rainfall deficits after moderate and large (VEI > 3) volcanic eruptions.

The global mean stratospheric aerosol loading has been modulated by a number of moderate volcanoes erupting after Mt. Pinatubo (June 1991)^24^. These volcanic eruptions also produced long-lasting perturbations in the global aerosol optical depth (AOD) and reflectivity^25^. The global aerosol radiative forcing and induced cooling from moderate volcanic eruptions since 2000 were estimated at −0.19 ± 0.009 W m^−2^ and −0.05 to −0.12 °C, respectively^26^. Past studies also show that surface cooling is known to cause a reduction in the Indian monsoon circulation^6^.

During the monsoon season, the localised aerosol layer over Asia known as the Asian tropopause aerosol layer (ATAL)^27–29^ has been shown to amplify the severity of Indian droughts^7^ by solar dimming. CALIPSO and SAGE-II satellite observations indicate that moderate-to-large volcanic eruptions, e.g. Kasatochi (August 2008), Sarychev (June 2009), Nabro (June 2011), have also been instrumental in enriching the ATAL^25,30^. These volcanic eruptions have enhanced the AOD in the lowermost stratosphere by ~ 30% of the global stratospheric aerosol optical depth^31^.

A further significant influence on the Asian summer monsoon and its association with volcanic eruptions and the ATAL is the El Niño Southern Oscillation (ENSO)^7,15,18^. Large volcanic eruptions can trigger atmospheric Kelvin waves and therefore shorten La Niña and lengthen El Niño periods within two years after the eruption^32^. El Niño periods are known to produce strong anomalous subsidence and a weakening of the Indian monsoon^4,33^. There are complex unexplored linkages of the Indian monsoon weakening and associated droughts with (1) aerosol layers in the UTLS, (2) volcanic eruptions thickening UTLS aerosol layers, and (3) the El Niño induced circulation which can be amplified by volcanic eruptions. Here we use a number of satellite observations (CALIPSO, MIPAS, MISR, TRMM) and climate model simulations (Max‐Planck‐Institute Earth System Model (*MPI*‐*ESM*) and ECHAM6-HAMMOZ) to investigate the role of moderate and large (VEI > 3) tropical volcanic eruptions in inducing drought conditions over India. The simulated rainfall is evaluated with the India meteorology department (IMD) and Global Precipitation Climatology Project (GPCP) rainfall data (see “Methods” section for details of data sets and methodology).

## Moderate and large volcanic eruptions and Indian droughts

**Figure 1:**
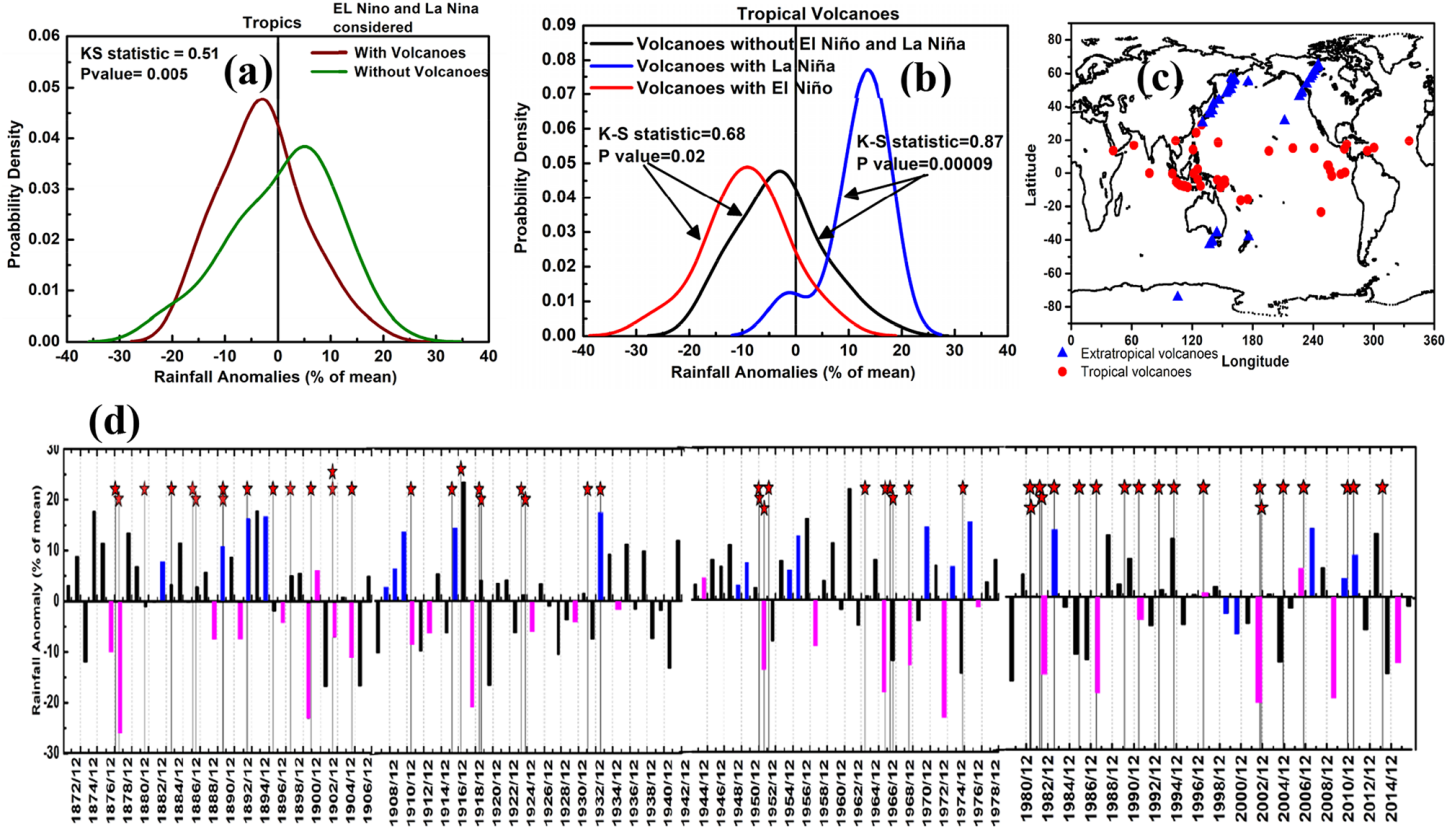
Volcanic eruptions and Indian summer monsoon rainfall: (**a**) probability distribution of rainfall anomalies within two years of eruption, when stratified with and without tropical volcanic eruptions; (**b**) probability distribution of rainfall anomalies within two years of a tropical volcanic eruption during El Niño, La Niña, and normal years; the statistical measures of K-S test shown in (**a**,**b**) indicates that distributions are distinct; (**c**) spatial distribution of moderate-to-large (VEI > 3) volcanic eruptions; (**d**) time series of June–September mean precipitation anomaly (%) during 1871 –2016 along with tropical (30° S–30° N) volcanic eruptions indicated with stars. Bars in magenta and blue in panel (**d**) indicate El Niño and La Niña years, respectively. Details of the volcanic eruption are listed in Table S1. Anomalies are obtained as difference in rainfall amount of the respective year and climatology of 1871–2016 (figure created using the COLA/GrADS software).

Figure 1a,b shows the probability distribution of observed ISMR anomalies during 1871–2016. The data indicate substantial rainfall deficits for monsoon seasons within two years after a moderate or large tropical volcanic eruption, compared to monsoon seasons without such an eruption in the preceding two years (Kolmogorov–Smirnov (K-S) test measure = 0.5, P values = 0.05 given in Fig. 1a indicates that the distributions with and without volcanoes are distinct. The K-S test measure and P-values shown in Fig. 1b and Fig. S1a,b also indicates that the corresponding distributions are distinct) (also see Table S1). Notably, this is in contrast to the observed ISMR anomalies during summer monsoon seasons following extratropical volcanic eruptions, which tend to show positive (wet) rainfall anomalies (Fig. S1a). This is likely due to the spatial distribution of the moderate and large (VEI > 3) volcanic eruptions. As shown in Fig. 1c, while tropical eruptions are mostly located near the maritime continent and the Niño-3 region, the extratropical eruptions are located near the north western Pacific and western Canada. However, if eruptions in the NH extratropics occur in June the Asian summer monsoon circulation transports volcanic aerosol into the tropics^34^. All five NH extra-tropical eruptions in June, Novarupta 1912, Komakatake 1929, Spurr 1992, Sheveluch 2001, and Sarychev 2009 (Table S2) are followed by a drought over the Indian region.

ENSO is also known to be an important factor contributing to Indian summer monsoon negative (El Niño) and positive (La Niña) rainfall anomalies^35^ (Fig. S1b). Several previous studies reported an El Niño signature in the Pacific after a volcanic eruption^32,36^. Figure 1b shows the probability distribution of ISMR anomalies corresponding to monsoon seasons following volcanic eruptions during the two phases of ENSO. Tropical volcanic eruptions associated with El Niño periods tend to produce Indian droughts while, volcanic eruptions associated with La Niña periods show positive rainfall anomalies (wet conditions). We find that 46 out of the 53 volcanic eruptions considered in our analysis (i.e. 87%) are followed by rainfall deficits during the 2-year period after the eruption. Of these 46 rainfall deficit periods, 36 occur during El Nino periods with 26 (26/53 = 49%) of them leading to droughts, i.e. rainfall deficit exceeding 10% of the seasonal climatological mean^37^ (Fig. 1d).

## Volcanic aerosols forming a blanket over the ATAL during the monsoon season

**Figure 2:**
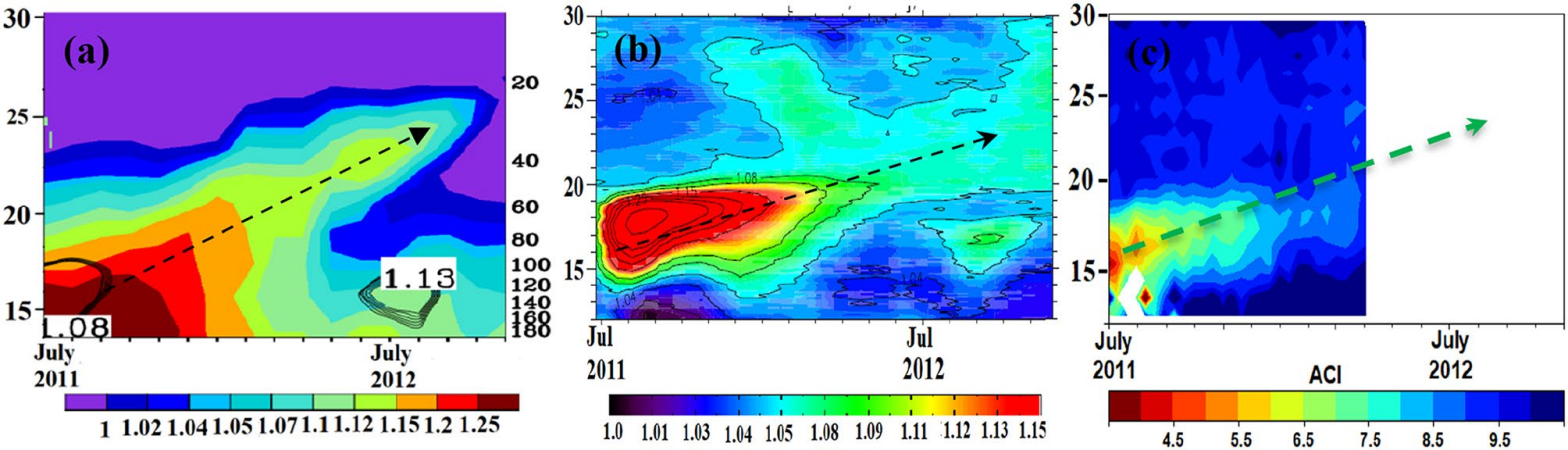
Aerosol vertical distribution during July 2011–November 2012 averaged over India (70–95° E; 10–30° N). Scattering ratio at 532 nm in the ECHAM6-HAMMOZ (Vol) simulation (**a**), Scattering ratio at 532 nm from CALIPSO (**b**), and Aerosol cloud index (ACI) estimates from MIPAS (**c**). Arrows indicate the transport of the NABRO plume. The ATAL is indicated as contours in (**a**,**b**) (Figure are created using the COLA/GrADS software and Fig. 2c is created using Python).

To further investigate the processes causing the impact of tropical volcanic eruptions on Indian monsoon rainfall, we focus on the Nabro, Eritrea (13.37° N, 41.7° E) volcanic eruption of 12–13 June 2011, a tropical moderate eruption, which injected 1.3–2.0 Tg of SO_2_ into the atmosphere^30,38^. Using the ECHAM6-HAMMOZ model volcano (Vol) simulation where 1.5 Tg of SO_2_ were injected at 42° E, 13° N on 12 June 2011 (details in “Methods” section) we show the vertical dispersion of the volcanic plume in Fig. 2a. Our simulations indicate that the Nabro volcanic plume formed a thick aerosol layer in the UTLS over the Indian region lasting up to October 2012 (Fig. 2a). The volcanic aerosol partially enters the monsoon anticyclone causing a thickening of the ATAL during monsoon 2011 (July–August-September). The aerosol backscatter ratio measured by CALIPSO and aerosol cloud index (ACI) derived from MIPAS also shows a similar dispersion of the volcanic aerosol plume (Fig. 2b,c). The aerosol is transported to higher altitudes and forms an additional layer above the ATAL during the subsequent monsoon in 2012 (the ATAL is indicated as contours in Fig. 2a,b). The aerosol backscatter ratio measured by CALIPSO and aerosol cloud index (ACI) derived from MIPAS measurements confirm the simulated dispersion of the volcanic aerosol plume (Fig. 2b,c). Both, the ECHAM6-HAMMOZ simulations and the CALIPSO data show the presence of two aerosol layers (ATAL and the volcanic layer) during the summer monsoon of 2012 (note there are no MIPAS data during this period). A previous study^30^ based on CALIPSO observations suggests that quasi-isentropic differential advection in the vertically sheared flow surrounding the Asian anticyclone helped in the formation of the stratospheric aerosol layer over the Asian monsoon region, while deep convection in the Asian monsoon played a minor role in transporting volcanic aerosols to the lower stratosphere. CALIPSO satellite measurements also indicate a diabatic ascent of the Nabro plume in the lower stratosphere at rates of 10 K month^−1^ for the first two months after the eruption and 3 K.month^−1^ after the dissipation of the Asian anticyclone^30^. Lidar observations in Tsukuba, Japan (36.05° N, 140.13° E), Saga, Japan (33.24° N, 130.29° E) and Wuhan, China (30.5° N, 114.4° E) also show significant vertical spread of Nabro aerosols^39^. Also, MIPAS satellite measurements and ground based lidar measurements in Europe show an increase of the aerosol layer thickness with time^40^. Raman Lidar measurements at Gwangju, Korea (35.10° N, 126.53° E) show that the geometric depth of the aerosol layer was 2–3 km in June 2011, which expanded to 10 km within the next two months^41^.

## Radiative impacts and surface cooling

Large volcanic eruptions are known to cause a substantial increase in the stratospheric aerosol layer which typically lasts for about 2–3 years^42,43^. These aerosol particles are more efficient at reflecting shortwave solar radiation than at attenuating longwave radiation emitted from the Earth’s surface, which results in a cooling of the troposphere and surface^44^. The El Chichón eruption in April 1982 produced a negative tropospheric radiative forcing (RF) of −2 to −4 Wm^−2^ over a year^45^ and the Pinatubo eruption in June 1991 produced a RF at the top-of-the-atmosphere (TOA) of approximately −4.5 Wm^−2^ over the region 40° S–40° N^44^. Stratospheric volcanic aerosols from 2008 to 2011 have been estimated to have imposed an aerosol RF of −0.11 (−0.15 to −0.08) Wm^−2^^46^.

Our ECHAM6-HAMMOZ simulations analysed here indicate that the NABRO eruption has elevated stratospheric (potential temperature > 350 K) AOD by 0.024 (50%) (from 0.048 in the CTL simulation to 0.072 in the Vol simulation) in July 2011 and by 0.0061 (14%) (from 0.044 in the CTL simulation to 0.05 in Vol simulation) in July 2012 over the Asian region (5–105° E; 15–45° N). A previous study^31^ has also reported an AOD enhancement of 0.01 in the stratosphere over the Asian monsoon region in July 2011 in comparison with the volcanically quiescent period 1997 –2000. CALIPSO satellite observations show AOD variation similar to ECHAM6-HAMMOZ, although the model overestimates AOD by 4–30% compared to CALIPSO (Fig. S2a)**.**

**Figure 3:**
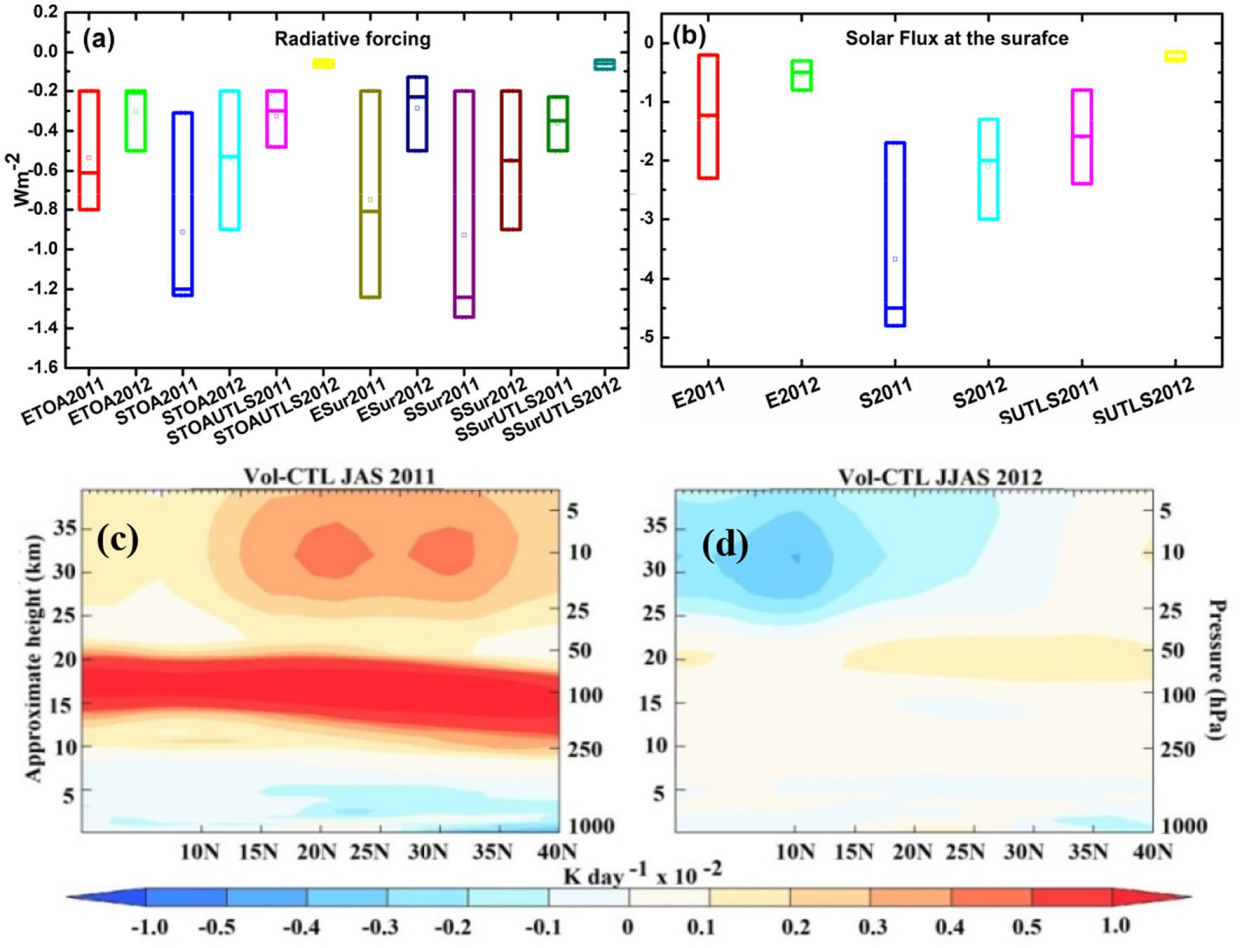
(**a**) Distribution of anomalies (Vol-CTL) in net direct radiative forcing at TOA (Wm^−2^) and the surface (Wm^−2^) for 2011 and 2012 monsoon from the ECHAM6-HAMMOZ simulations (ETOA and ESur), SOCRATES model (STOA and SSur), aerosols in the UTLS from SOCRATES: STOAUTLS and SSurUTLS), (**b**) same as (**a**) but changes in net solar radiation (flux) at the surface (W.m^−2^) (ECHAM6-HAMMOZ—E2011 and E2012; SOCRATES—S2011 and S2012, aerosols in the UTLS from SOCRATES—SUTLS2011 and SUTLS2012). Bars in panels (**a**) and (**b**) correspond to minimum, mean, maximum values. Vertical distribution of changes in heating rate (K day^−1^) averaged for 70°E–95°E and monsoon (**c**) 2011, and (**d**) 2012. (Figure created using the COLA/GrADS software).

The estimates of seasonal mean radiative impacts of the Nabro aerosol are provided for two subsequent monsoon seasons, July–September 2011 (June is not considered since the Nabro plume entered the anticyclone ~ 26 June 2011) and June–September 2012, referred hereafter as monsoon 2011 and monsoon 2012 respectively. Estimates of mean RF over the Indian region (75–90° E; 10–28° N) obtained from ECHAM6-HAMMOZ simulations (Vol-CTL) suggest that the Nabro aerosol layer produced negative RFs in 2011 monsoon (−0.81 Wm^−2^ at the surface; −0.61 Wm^−2^ at TOA) and in 2012 monsoon (−0.23 Wm^−2^ at the surface; −0.21 Wm^−2^ at TOA) (Fig. 3a). Using the SOCRATES model^47,48^ to isolate just the aerosol radiative effect (i.e. without accounting for other associated changes, e.g. dynamics), we estimate the following direct aerosol radiative forcing (Vol-CTL): −1.34 Wm^−2^ at the surface and −1.23 Wm^−2^ at TOA in 2011 monsoon, and −0.55 Wm^−2^ at the surface and −0.53 Wm^−2^ at TOA in 2012 monsoon (Fig. 3a). The SOCRATES model also allows us to quantify the contribution of the simulated enhanced Nabro volcanic aerosol in the UTLS (150–30 hPa): −0.35 Wm^−2^ at the surface and −0.3 Wm^−2^ at TOA in 2011 monsoon, and at −0.06 Wm^−2^ at the surface and −0.06 Wm^−2^ at TOA in 2012 monsoon (Fig. 3a). A previous study^30^ based on CALIPSO observations shows that the increase in Nabro aerosols (at 14–40 km) has imposed a RF at the TOA −0.8 W.m^−2^ to −0.5 W.m^−2^ over South Asia in July and August 2011 (a local peak of −1.6 W.m^−2^ July 2011)^30^ along with a global mean RF changes −0.3 Wm^−2^ during 2011–2012^31^.

Our ECHAM6-HAMMOZ model simulations show that the volcanically enhanced sulphate aerosol layer has led to a reduction in the solar flux reaching the surface over India (75–90° E; 10–28° N) by −1.23 W.m^−2^ and −0.50 W m^−2^ in the monsoon season of 2011 and 2012, respectively (Fig. 3b). The corresponding changes in solar flux at the surface estimated using the SOCRATES radiation model (i.e. simulating only the volcanic sulphate aerosol direct radiative effect) are −4.5 W m^−2^ and −2.0 W m^−2^ in the monsoon season of 2011 and 2012, respectively. The contribution of the volcanic aerosol layer in the UTLS (150–30 hPa) quantified with the SOCRATES radiation model is a reduction in surface solar flux of −1.59 W m^−2^ and −0.27 W m^−2^ in the monsoon seasons of 2011 and 2012, respectively (Fig. 3b).

Previous studies show that large volcanic eruptions can produce a global cooling at the surface for typically 2 to 3 years after the eruption^49,50^. Ocean–atmosphere climate model simulations show that an increase in the moderate volcanic activity during 2003–2012 has led to a reduction in the global mean warming trend of 0.08 °C in ten years^50^. Our MPI-ESM simulations, which couples the atmosphere, ocean and land surface (details in “Methods” section) show that the Nabro volcanic sulphate aerosol over North India (75–90°E, 20–35°N) has produced a mean cooling of −0.055 °C (−0.08 to −0.03 °C) in 2011 monsoon and −0.075 °C (−0.09 °C to −0.06 °C) in 2012 monsoon. Dynamical changes and subsidence associated with El Niño might have caused higher cooling in the monsoon season of 2012 than in 2011. This cooling is a factor of 10 smaller than the cooling caused by the Mt Pinatubo eruption, which amounts to −0.6 °C to −0.5 °C global mean surface temperature change during 1992–1993^51^. Observations suggest that large volcanic eruptions for the last 150 years have produced a global mean surface cooling of 0.3°C^52^. Temperature records for the past 450 years from corals, tree rings and ice cores show that volcanism in the tropics has produced a cooling of −0.1 °C in the tropics^53^.

Using the SOCRATES model we estimated the changes in heating rates induced by the Nabro volcanic aerosol. As shown in Fig. 3c, increases in heating rates of 0.01 K.day^−1^ at ATAL altitudes (15–20 km) and 0.003–0.005 K.day^−1^ above ATAL altitudes (20–35 km) are simulated during the 2011 monsoon season, in conjunction with some small reductions of −0.001 to −0.002 K.day^−1^ at lower altitudes in the troposphere. This is driven by: (i) a local longwave (LW) heating, due to strong absorption of LW radiation by the aerosol; (ii) some LW heating below the aerosol layer and some LW cooling above the aerosol layer; (iii) short wave (SW) heating above the aerosol layer, due to scattered SW radiation from the aerosol layer being reflected upwards and absorbed by radiatively active gases (e.g. ozone) in that region and direct radiative heating from SW absorption by the aerosol itself; and (iv) SW cooling below the aerosol layer, due to the aerosol scattering SW radiation and therefore less SW radiation reaching those lower levels of the atmosphere. Substantially smaller changes are simulated during the monsoon season of 2012 (Fig. 3d) due to the much smaller aerosol loadings and their location at higher altitudes. A thicker and broader ATAL over the Indian region due to aerosol enhancement during El Niño was shown to lead to a reduction of solar flux of up to −5 Wm^−2^ and negative heating rate anomalies of up to −0.05 K.day^−1^ over North India^7^.

## Reduction in Indian summer monsoon precipitation

**Figure 4:**
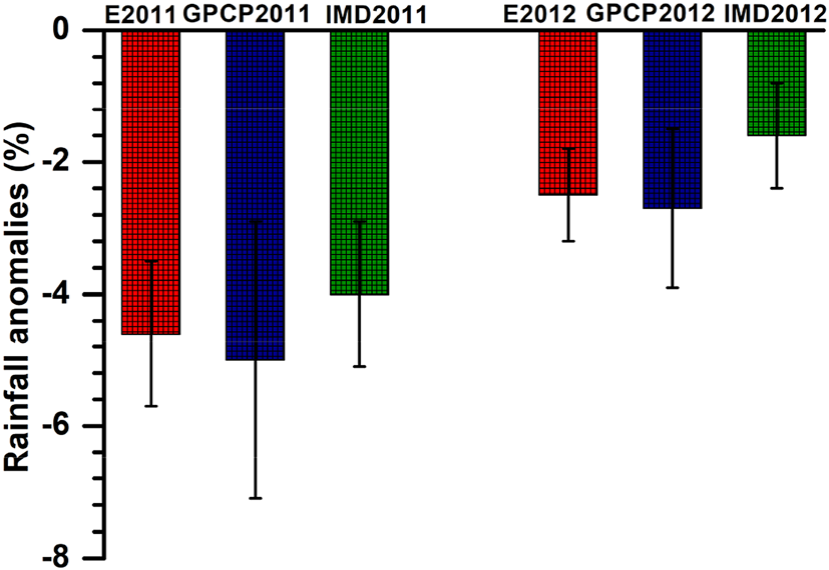
Distribution of anomalies (Vol-CTL) of rainfall (%) (averaged for 78–93°E, 8–35°N and for monsoon 2011 (July–September) and monsoon 2012 (June–September) from MPI-ESM represented as E2011, E2012; Global Precipitation Climatology Project (GPCP) (precipitation of monsoon 2011/2012—climatology of 1981–2015), India Meteorology Department (IMD) data (precipitation of monsoon 2011/2012—climatology of 1950–2015) (Figure created using Origin (OriginLab, Northampton, MA)).

MPI-ESM simulated anomalies in precipitation (Vol-CTL) indicate that volcanic aerosols injected by the Nabro eruption have induced a reduction precipitation over the Indian region by −4.6% (−4.85 mm day^−1^) in 2011 monsoon and −2.5% (−3.12 mm day^−1^) in 2012 monsoon (Fig. 4). Precipitation reductions by −5.0% in 2011 monsoon and −2.7% in 2012 monsoon in comparison with the climatology (1981–2015) are also evident from the Global precipitation climatology project (GPCP) analysis of precipitation. India Meteorological Department (IMD) precipitation data also show a reduction in precipitation of −4.0% in 2011 monsoon and −1.1% in 2012 monsoon compared to the climatology (1950–2015) (Fig. 4). The weekly departures of precipitation changes (deviation from normal rainfall) of these measurements, when averaged over the Indian region, further show reduction in rainfall in 2011 and 2012 monsoon (Fig. S2b,c). However, the Tropical Rainfall Measuring Mission (TRMM) rainfall data show an increase in precipitation of 4.0% in 2011 and a reduction of −0.9% in 2012 in comparison with the climatology (1998–2015) (Fig. 4). It should be noted however that TRMM data have a substantial bias (65%) over the Indian land mass in comparison with IMD rain gauge data^54^*.* Also, TRMM data show large biases over the Himalayas in comparison to India meteorological rain gauge data^55^. We have also obtained changes in rainfall in the 2011 and 2012 monsoon season in comparison with the climatology (1998–2015) over the Indian region, which excludes the Himalayas and Western Ghats (80E—93° E; 8 N–27° N). This region shows a reduction in rainfall in the monsoon season of 2011 (−0.59%) and 2012 (−4.2%). Importantly, the MPI-ESM simulations captured the rainfall reduction in the monsoon seasons of 2011 and 2012.

**Figure 5:**
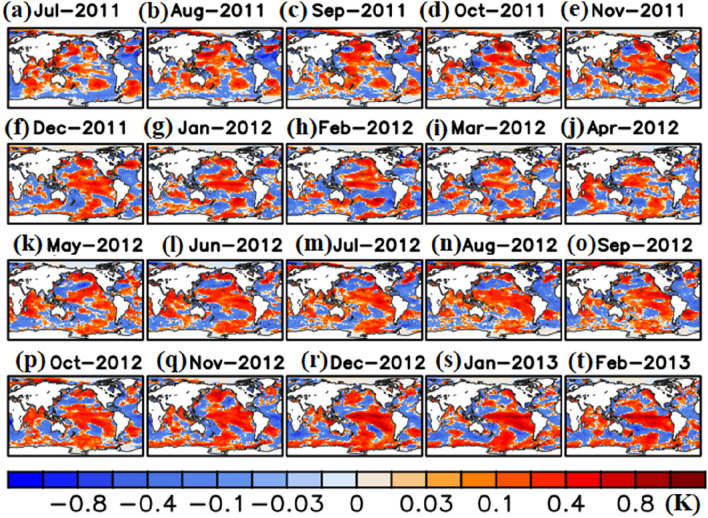
Anomalies (Vol-CTL) in temperature of sea water (K) from MPI-ESM (**a**)-(**t**) from July 2011 to February 2013. (Figure created using the COLA/GrADS software).

Thus, our model simulates volcanic aerosol induces El Niño like warming in the central Pacific in 2011 (reduced La Niña signal) and in 2012 (strengthened El Niño condition) (Figs. 5a–t, S3) and El Niño is known to cause reduction in ISMR^4^ (discussed in “Association of volcanic eruptions with ENSO” section). The precipitation reduction is higher in the 2011 monsoon season than in 2012. During 2011, the Nabro volcano injected aerosols into the monsoon anticyclone, which formed a thicker aerosol layer extending from the upper troposphere to the lower stratosphere (150 hPa to 30 hPa) over the Indian region. In contrast, in 2012, the volcanic aerosol layer was thinner and located in the lower stratosphere above the thin ATAL. A thicker aerosol layer in 2011 results in a stronger reduction of solar insolation reaching the surface than in 2012, the stronger negative radiative forcing in 2011 prompts a stronger tropospheric cooling in 2011 than the thin layer in 2012 (as discussed in “Radiative impacts and surface cooling” section). Thus, the precipitation decrease in 2012 can for the most part be attributed to the anomalous subsidence because of El Niño. Here, the thin layer of aerosol located in the lower stratosphere in 2012, has less effect on solar inhibition. Thus solar dimming by the thicker aerosol layer (extending from 150 to 30 hPa) in the year of eruption causes a higher precipitation decrease than the following El Niño year (2012).

Sulfate geoengineering studies also show a reduction in Indian summer monsoon precipitation due to aerosol dimming and dynamical changes caused by stratospheric heating induced by the injected aerosol^19,21^, e.g. an 8.5% (0.53 mm.day^−1^) reduction in precipitation was reported^21^. Our MPI-ESM simulations show that volcanic sulfate aerosols cause stratospheric heating and tropospheric cooling, which further leads to dynamical changes resulting in the precipitation reduction in 2011 and 2012. These are due to (1) the anomalous reversal of the monsoon Hadley circulation (Fig. S4a,b), which is also seen in NCEP data (Fig. S4c,d), (2) weakening of the low-level jet (Fig. S4c,d), (3) enhanced outgoing longwave radiation (OLR) (Fig. S4e,f) and (4) enhanced stability in the upper troposphere (Fig. S4g,h).

## Association of volcanic eruptions with ENSO

Stratospheric aerosols from explosive tropical volcanic eruptions are known to cause an anomalous surface cooling within two years following the eruption. This cooling can induce atmospheric Kelvin waves and drive equatorial westerly wind anomalies over the western Pacific, thereby favouring El Niño conditions and shortening La-Niña periods^32^. Further, an El Niño signature after volcanic eruptions has been reported by several previous studies^8,32^. Also, 350 years of records show a large number of El Niño episodes associated with volcanic eruptions^56^.

El Niño is one of the most important atmospheric phenomena causing Indian droughts^4^. The Hadley Centre Sea Ice and Sea Surface Temperature (HadISST) data indicate that while 2011 was a moderate La Niña year, there was a clear El Niño signal in 2012 (Fig. S3). CMIP5 simulations show that a volcanic forcing tends to favour an anomalous central Pacific warming that peaks during the year of the eruption and therefore produces an El Niño like signal irrespective of the ENSO preconditioning^32^. Our MPI-ESM simulation (Vol-CTL) for the Nabro eruption further confirms warming in the central Pacific in 2011 and 2012 (Fig. 5). The warming was relatively weak during in 2011 and 2012 monsoons. It further strengthened in the winter season in 2011 and 2012 (from November 2011 to January 2012 and December 2012—February 2013) (Fig. 5). The warming induced by the Nabro eruption weakens the La-Niña condition in 2011 and strengthens the El Niño in 2012 (Figs. 5 and S3).

**Figure 6:**
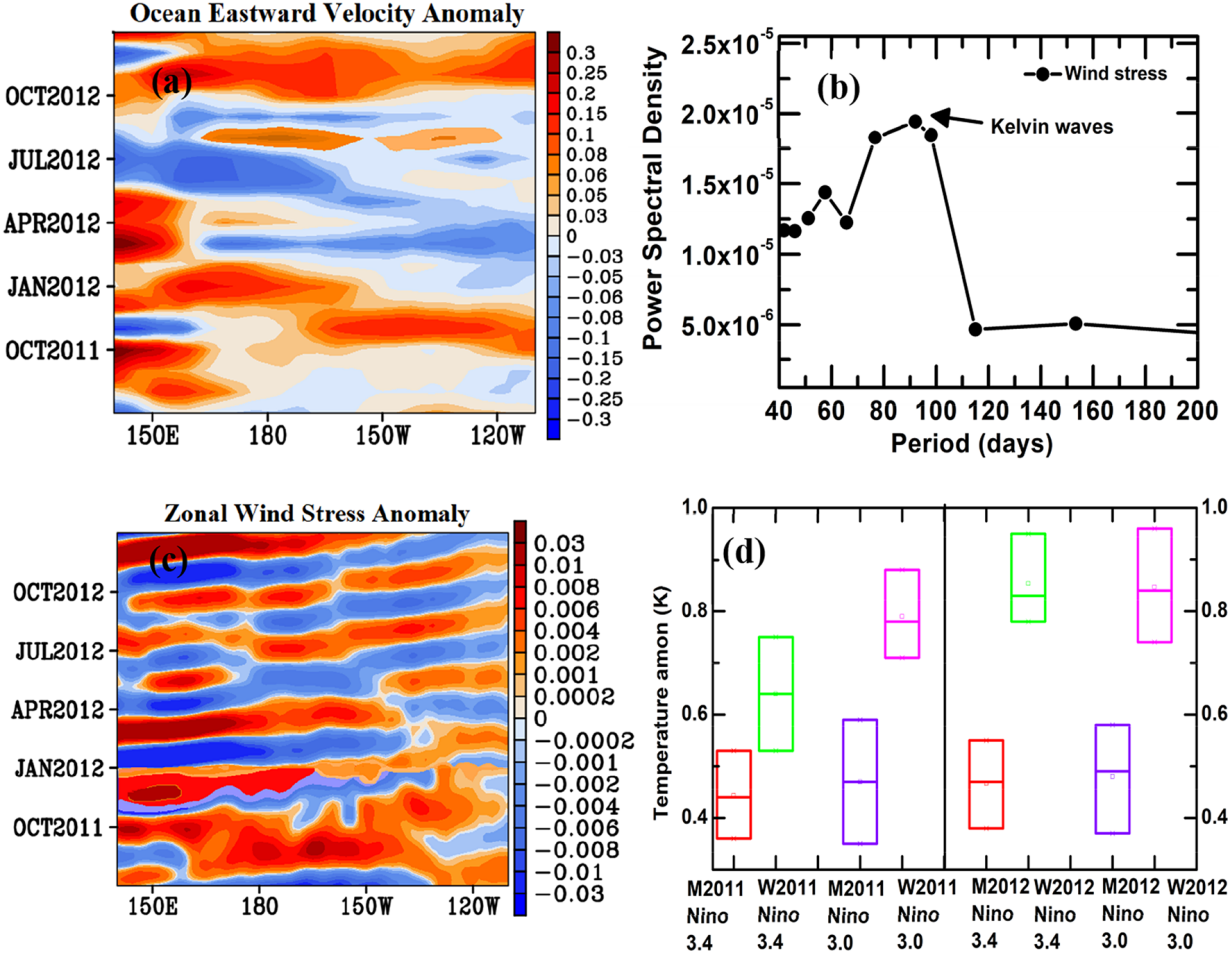
(**a**) Anomalies (Vol-CTL) in ocean eastward velocity (m s^−1^) averaged for 2°S–2°N, (**b**) Power spectral density plot of anomalies (Vol-CTL) in zonal wind stress (Pa) averaged for 2°S–2°N and 140° W, it indicates the Kelvin waves with dominant periodicity of 70–100 days during July 2011 to January 2013, (**c**) Eastward propagating Kelvin waves near the equator (averaged for 2°S–2°N) after application of band pass filter (70–100 days) on anomalies (Vol-CTL) in zonal wind stress (Pa), (**d**) Seasonal mean anomalies (Vol-CTL) in surface temperature of sea water (K) at the Niño 3 (5°S to 5°N, 150 W and 90 W) and Niño 3.4 (170°W to 120°W, 5°S to 5°N) regions. M2011 represents as 2011 monsoon (JAS 2011), W2011 as 2011 winter (December 2011-Feb2012), M2012 as 2012 monsoon (JJAS 2012), and W2012 as 2012 winter (December 2012-Feb2013). Bars in panel (**d**) correspond to minimum, mean, maximum values. Figure (**a**–**d**) are from the MPI-ESM model simulations (Figure created using the COLA/GrADS software).

Our analysis indicates that the surface cooling over the Indian region induced by the layer of Nabro aerosols caused an atmospheric westerly wind anomaly in the central Pacific in July 2011 after the eruption (Fig. 6a). This wind anomaly resulted in downwelling equatorial oceanic Kelvin waves through air-sea interactions and eventually drove a surface warming in the central Pacific during July 2011 to February 2013 (Fig. 6b–d). This is consistent with analysis from CMIP5 simulations that showed how large volcanic eruptions can induce cooling in tropical Africa, i.e. the volcanically induced atmospheric cooling produced a Kelvin wave, driving an El Niño like warming in the Pacific^32^. Interestingly, El Niño like warming by Kelvin wave dissipation is stronger in the second year after the eruption^32^. In agreement to this, our model simulations (Vol-CTL) also show warming due to Kelvin wave dissipation triggered by the eruption (Niño 3.0 and Niño 3.4 regions) is stronger in the following year (monsoon and winter seasons in 2012) than the year of volcanic eruption (monsoon and winter seasons in 2012) (Figs. 5, 6d). A clear signal of El Niño like warming in 2011 and 2012 is also seen in Fig. 6d, it weakens La Nina features in 2011 and strengthens the El Nino features in 2012 (Fig. S3).

El Niño is known to induce anomalous large scale subsidence over the Indian region associated with a subdued precipitation^4^. The simulated (Vol-CTL) effect of the volcanic eruption on the regional circulation shows subsidence associated with a descending branch of the Walker circulation over the Indian region during the 2011 and 2012 monsoons (Fig. S5a,b). This is caused by the volcanically induced warming in the central Pacific. The model simulations also show a weakening of the Hadley circulation in monsoon seasons of 2011 and 2012 (Fig. S4a,b) in agreement with reanalysis data (Fig. S4c,d). Thus we can infer the role of volcanically induced El Niño like warming in the central Pacific inducing the subsidence over the Indian region during the 2011 and 2012 monsoons.

**Figure 7:**
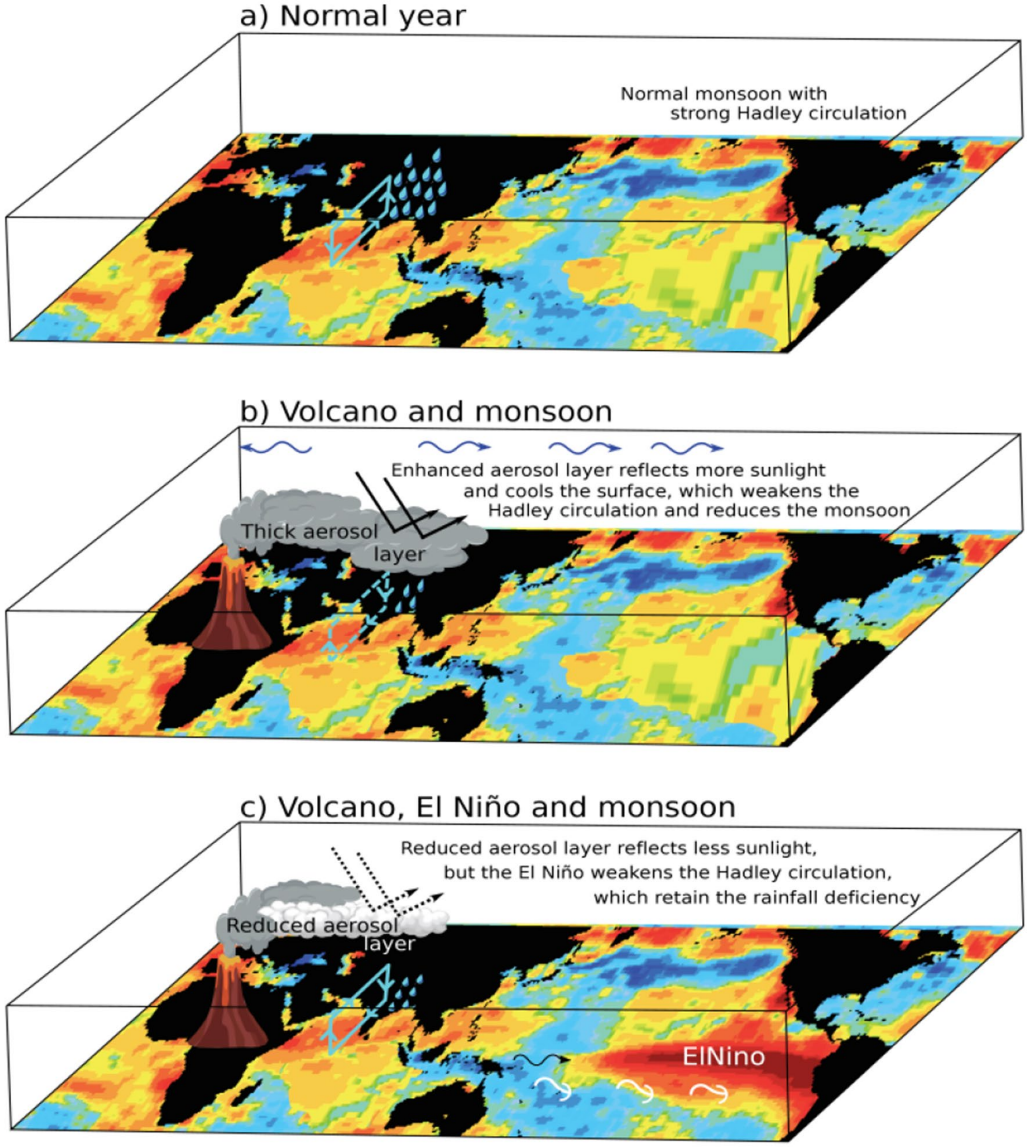
A schematic of the impact of volcanic eruptions on monsoon precipitation: (**a**) normal monsoon with a strong Hadley circulation, (**b**) Nabro volcanic aerosol leading to a thick aerosol layer in the UTLS comprising of the ATAL, which reflects more solar radiation leading to a weak Hadley circulation (anomalous; denoted with sign reversal and dotted lines) and a reduction in rainfall. (**c**) The tropospheric cooling induced by the thicker ATAL (as in volcano year, "**b**") induces an El Niño in the following year (through anomalous atmospheric Kelvin and Rossby waves (shown as a blue wave in “**b**”), which induce a westerly wind burst (black wave) and produce an El Niño through downwelling Kelvin waves (shown in white colour)). However, the reduced aerosol layer lets pass more sunlight compared to the year of the eruption, but the El Niño weakens the Hadley circulation (anomalous; shown in lines [since it is less reduction than in the year of the eruption] with the inverted arrow) and retains the deficit rainfall condition. The SST patterns intend to indicate the normal and El Niño condition over the Pacific, rest of the oceanic basins are kept the same in all maps for illustration purpose (and are not connected with our simulations). Maps are prepared using NCL and 3D impact and schematic structures made utilizing Adobe illustrator.

Data records show that 49% (26 out of 53) of the moderate to large tropical volcanic eruptions during 1871–2016 have been associated with El Niño conditions within two years after the eruption and have caused droughts over India (Fig. 1 and Table S1). We argue that these moderate to large volcanic eruptions are linked to a reduction in monsoon precipitation for two consecutive years through a series of connected mechanisms: (i) thickening of the ATAL over India; (ii) formation of a thick layer of volcanic aerosol above the ATAL extending to the stratosphere, i.e. a double blanket; (iii) this double blanket effect due to additional volcanic plumes weakens the monsoon circulation via dynamical changes caused by the stratospheric heating and by its radiative cooling effect in the troposphere; (iv) the tropospheric cooling induces atmospheric Kelvin waves, which lead to warming in the central Pacific through air-sea interaction; (v) it also reduces La Niña features (e.g. following the Nabro eruption in 2011) and strengthens El Niño like conditions (e.g. in 2012), (vi) the El Niño favoured subsidence further exacerbates drought conditions in India (see Fig. 7). The precipitation reduction is higher in the year of the eruption due to the formation of a thick aerosol layer extending from the upper troposphere to the lower stratosphere in addition to the subsidence associated with the El Niño like warming. The year following the year of eruption continues as a drought year because of the stronger El Niño like warming compared to the previous year.

## Summary

This study highlights the role of tropical volcanic eruptions in enhancing the aerosol loading in the UTLS and reducing the Indian monsoon precipitation. This reduction occurs due to solar dimming and dynamical interactions through the weakening of the monsoon Hadley circulation and the low-level monsoon jet as well as by enhancing the stability of the upper troposphere. Volcanic eruptions may further cause a reduction of La Niña signals or induce an El Niño signal over the tropical Pacific, thus reducing the Indian summer monsoon precipitation. The changes in the aerosol loading in the UTLS induced by volcanic eruptions resemble to a certain extent those caused by intentional injections of stratospheric aerosol (or aerosol precursors) to compensate for atmospheric CO_2_ increase^19,21^. Our results are thus also relevant for assessing the impact of solar radiation management (SRM) geoengineering, which could exacerbate droughts in the Indian region. Further, an increasing trend in anthropogenic SO_2_ emissions (~ 4.8% per year) over the South Asian region due to enhanced industrialisation and biomass burning also leads to an increase in sulphate aerosols, which are transported to the lower stratosphere by the Asian monsoon convection^57^. This impact of increasing anthropogenic aerosol is also enhanced intermittently by episodic volcanic eruptions, which are injecting aerosol precursors and sulphate aerosols directly into the lower stratosphere. Such eruptions lead to large aerosol loadings in the lowermost stratosphere, with an important associated contribution to radiative forcing^31^. Satellite measurements (CALIPSO, SAGE II, GOMOS, and OSIRIS) confirm the rising trend in the stratospheric aerosol loading during 2002–2010, which is mainly driven by a series of moderate but increasingly intense volcanic eruptions primarily at tropical latitudes^25^. There are ~ 1500 active volcanoes worldwide^58^, including Asia (253), Japan (100), Africa (152), the maritime continent (127), United States of America (71), and Canada (21) (https://www.volcanodiscovery.com). They can significantly amplify the dimming already caused by the anthropogenic aerosol concentration over the South Asian monsoon region, which will affect the hydroclimate of the Asian region. Hence, along with other key factors, tropical volcanic eruptions are important contributors influencing Indian droughts with substantial socio-economic implications.

## Methods

### Model description and experimental setup

To understand the impact of the Nabro volcanic aerosol on Indian summer monsoon precipitation we employed a two-step method, where the radiative properties of the aerosol plume were first simulated with an aerosol-chemistry-climate model and then implemented as prescribed fields into a state-of-the-art Earth System Model. The ECHAM6-HAMMOZ aerosol-chemistry-climate model was used for the Nabro volcanic plume simulations. The model comprises of an atmospheric general circulation module (ECHAM6.3), a tropospheric chemistry module (MOZ1.0), and an aerosol module, namely the Hamburg Aerosol Model (HAM2.3)^59,60^. The model parametrisation and other details are documented in previous studies^57^. Aerosol microphysics is simulated by the Sectional Aerosol module for Large Scale Applications (SALSA) module^61^, where the aerosol size distribution is described by 10 size sections for soluble and 7 size sections for insoluble particles. The model simulations were performed at a spectral resolution T63 corresponding to 1.875° × 1.875° in horizontal and 47 vertical levels from the surface to 1 hPa with a time step of 20 min. The anthropogenic and fire emissions of sulfate, black carbon (BC) and organic carbon (OC) are based on the AEROCOM-ACCMIP-II emission inventory^62^. We performed Nabro volcano and control simulations starting from 1 to 10 January 2011 to obtain a 10-member ensemble mean. These simulations end on 31 December 2013 (Table S3). In all 10 members of Vol simulations 1.5 Tg of SO_2_ was injected at 42° E, 13° N on 12 June 2011. The plume was equally distributed between 10 and 17 km*.* It should be noted that the anthropogenic aerosols are the same in the Vol and CTL simulations. Therefore the difference in radiative effect obtained from Vol and CTL simulations correspond to volcanic aerosol effects.

To estimate rainfall we performed experiments with the MPI-ESM^63^. This model couples the atmosphere, ocean and land surface through the exchange of energy, momentum, and water, which allows changes in SSTs, temperature of sea water and signature of El-Niño and La Niña to be estimated. The model consists of the atmospheric general circulation model ECHAM6^64^ and the MPI Ocean Model (MPI-OM^65^). MPI-OM applies a conformal mapping grid with a horizontal resolution ranging from 22 to 350 km. The ocean model includes a Hibler-type dynamic–thermodynamic sea ice model with viscous–plastic rheology^66^. Ocean and atmosphere are coupled daily without flux corrections using the Ocean–Atmosphere Sea Ice Soil, version 3 (OASIS3) coupler^67^. The version of MPI-ESM used in this study does not simulate aerosols explicitly. To study climate impacts of the Nabro volcanic eruption, the aerosol properties from ECHAM6-HAMMOZ simulations were implemented to MPI-ESM as prescribed fields. The method is the same as^68^. The aerosol optical depth, single-scattering albedo, and the asymmetry factor were archived for 14 shortwave bands and absorption AOD in 16 longwave bands of radiative transfer model of ECHAM6-HAMMOZ. Then the aerosol radiative properties were implemented as 3D fields to MPI-ESM as monthly ensemble mean values. To include only upper tropospheric and stratospheric aerosols from the Nabro volcanic eruption, the lowest 14 model level (up to 4–6 km altitude) of aerosols fields of ECHAM6-HAMMOZ simulations were excluded and default MPI-ESM tropospheric aerosols^69^ were used instead. The same T63L47 atmospheric resolution was used in MPI-ESM simulation, as in ECHAM6-HAMMOZ. Simulations were based on Representative Concentration Pathway 4.5^70^ scenario and started from the year 2011 and continued until the end of the year 2013. Ten ensemble members were simulated to both CTL and Vol scenarios (Table S3).

The offline model estimates for the volcanic sulphate aerosol direct radiative forcing and associated changes to heating rates were calculated with the SOCRATES radiative transfer model^47,48^ with 6 shortwave and 9 longwave bands, using the CLASSIC aerosol scheme^71^.

### Observations and reanalysis data

Aerosol Optical Depth (AOD) measurements from two satellites (1) Multi-Angle Imaging Spectroradiometer (MISR) (https://misr.jpl.nasa.gov/getData/accessData/)^72^, and (2) CALIPSO^27^ (https://misr.jpl.nasa.gov/getData/accessData/), from July 2011 to December 2012 were used in this study. (https://eosweb.larc.nasa.gov/project/calipso/calipso_table).

We used zonal and meridional wind data from the National Centre for Environmental Prediction (NCEP) reanalysis, available for the period 1948–2016. Rainfall data sets used are from India Meteorology department (IMD) for the period 1871–2016, Tropical Rainfall Measuring Mission (TRMM) daily rainfall data (3B42) for the period 1998–2015 (https://gpm.nasa.gov/data-access/downloads/trmm) and Global Precipitation Climatology Project (GPCP) containing data from rain gauge stations, satellites, and sounding observations for the period 1981–2015 (https://climatedataguide.ucar.edu/climate-data/gpcp-monthly-global-precipitation-climatology-project). Hadisst data were used for the period 1980–2016 (https://climatedataguide.ucar.edu/climate-data/sst-data-hadisst-v11). We considered the occurrence of strong El Niño during the years 1876, 1877, 1888, 1891, 1896, 1899, 1900, 1902, 1904, 1911, 1913, 1918, 1925, 1930, 1935, 1944, 1951, 1957, 1965, 1968, 1972, 1976, 1982, 1987, 1991, 1997, 2002, 2006, 2009 and 2015. The large volcanic eruption (VEI ≥ 3), the El Niño condition following within two years and the Indian droughts are tabulated in Tables S1 and S2. In our analysis, we considered a monsoon season of the co-occurring El Niño year since correlations between Indian summer monsoon rainfall and Niño-3.4 index exhibits a maximum negative correlation for a zero-lag year^73^.

### Evaluation simulated precipitation with rain gauge measurements

Figure S6a–h shows distributions of simulated precipitation (MPI-ESM Vol simulation), IMD measurements, TRMM satellite retrievals, and GPCP data for the monsoon in 2011 (July –September) and 2012 (June–September). There is large spatial variation in precipitation among the data sets although all data sets show similar spatial patterns (e.g., higher rainfall at Southern slopes of Himalayas, Western Ghats and Indo Gangetic plains). We quantify the difference in simulated precipitation with respect to IMD, and GPC data over India (78–93°E, 8–35°N) (see Fig. S6i). The model underestimates precipitation compared to IMD by 0.9 mm.day^−1^ (Vol: 7.6 mm.day^−1^; IMD: 8.5 mm.day^−1^) in 2011 and 1.0 mm.day^−1^ (Vol: 7.0 mm.day^−1^, IMD: 8.0 mm.day^−1^) in 2012. Also, simulated rainfall is higher than GPCP by 1.1 mm.day^−1^ (Vol: 8.5 mm.day^−1^, GPCP: 7.4 mm.day^−1^) in 2011 and by 1.3 mm.day^−1^ (Vol: 8.0 mm.day^−1^, GPCP: 6.7 mm.day^−1^) in 2012. Figure S6i shows that the model underestimates rainfall in comparison with IMD and overestimates GPCP over the Indian landmass.

At the Southern slopes of the Himalayas the model overestimates precipitation by 2.3 mm.day^−1^ than GPCP and at Western Ghats it underestimates by 6.2 mm.day^−1^ in comparison with IMD and by 4.1 mm.day^−1^ in comparison with GPCP. These model deficiencies may be due to the fine orthography of these regions which is not well represented in MPI-ESM due to its coarse resolution. An accurate monsoon simulation is still a challenge even with high-resolution climate models^2^**.** There are uncertainties in the model due to transport processes, employed emission inventory, and various parametrisations^7,57^. Importantly, there are differences between the observational data sets, IMD, and GPCP as well. These differences may be due to different techniques of measurements, while IMD uses a network of rain gauge station measurements and GPCP combines measurements from rain gauge stations, satellites, and sounding observations. However, the limitations for the model results are the same in CTL and Vol simulations and generate monsoon precipitation reduction (Vol-CTL) (Fig. S7). Similarly IMD and GPCP also show precipitation reduction over the Indian region in monsoon 2011 and 2012 (Fig. S7). Considering fair performance of the model in simulating precipitation over India we proceed carefully employing model results for understanding the impact of the Nabro volcanic eruption in Indian summer monsoon precipitation.

### Evaluation of NABRO AOD and plume

Figure S8a–d shows the distribution of total AOD from MISR observations and ECHAM6-HAMMOZ (Vol) simulations. It shows that the model could simulate the spatial pattern of high amounts of aerosols over the Indo-Gangetic Plain and Mongolia desert, although the AOD is underestimated by ~ 0.07 in the model.

The comparison of the simulated enhancement of AOD with observations at various locations also shows reasonable agreement. Our simulations show an enhancement of AOD in the UTLS in a grid at Eritrea of ~ 0.08 on 25th June 2011, which is in agreement with observations, e.g., lidar measurements in Korea, Japan, and China that show an increase in UTLS AOD of ~ 0.07 during June 2011^39,40^. An increase in AOD ~ 0.012–0.03 between 12 and 20 km over Asia has also been reported in the past^41^ using CALIPSO measurements during 16–31 July 2011. CALIPSO satellite observations also show an increase in global mean AOD by ~ 0.01 in the UTLS in June 2011 due to the Nabro eruption^31^.

Further, we analyse the dispersion of aerosols in the UTLS. Figure S9a–d shows anomalies of sulfate aerosols (Vol-CTL) depicting the progression of simulated sulfate aerosol plume during 14–20 June 2011 at an altitude of 12–16 km. Figure S9a shows that on 14 June the sulphate aerosol plume was entrained in the south westerly flow over the Middle East on the northwest. On 20th June 2011, the plume circumnavigated the Asian anticyclone (Fig. S9d). The aerosol concentration is mostly located in the northern hemisphere. Aerosol measurements from the MIPAS satellite on the same days, filled with Lagrangian trajectory traces calculated by MPTRAC from measurements within ± 3 days driven by ERA5 data, indicate a similar progression of the NABRO plume during 14 –20 June (Fig. S9e–h). A similar distribution and progression of the Nabro plume is seen in CALIPSO and AIRS observations^30^. The simulated zonal spread of the aerosol in the UTLS (16–20 km) also shows reasonable agreement with the aerosol observations from MIPAS satellite (Fig. S9i–l).

## Supplementary information

Supplementary Information.
