# Supplementary material for: The role of tropical volcanic eruptions in exacerbating Indian droughts
Source: Sci Rep. 2021 Feb 1;11:2714. doi: 10.1038/s41598-021-81566-0 (PMC7851170; doi:10.1038/s41598-021-81566-0)
Supplement: Supplementary file 1 — Supplementary Information. [file 41598_2021_81566_MOESM1_ESM.pdf]

The role of tropical volcanic eruptions in exacerbating Indian droughts

Suvarna Fadnavis[1*], Rolf Müller[2], Tanusri Chakraborty[1], Sabin, T.P.[1], Anton Laakso[3], Alexandru Rap[4], Sabine Griessbach[5], Jean-Paul Vernier[6,7], Simone Tilmes[8]

[1]Indian Institute of Tropical Meteorology, MoES, India

[2] Forschungszentrum Jülich GmbH, IEK7, Jülich, Germany.

[3] Finnish Meteorological Institute, Kuopio, Finland

[4] School of Earth and Environment, University of Leeds, Leeds, UK

[5]Forschungszentrum Jülich GmbH, Jülich Supercomputing Center, Jülich, Germany

[6]National Institute of Aerospace, Hampton, Virginia, United States.

[7]NASA Langley Research Center, Hampton, Virginia, United States.

[8]National Center for Atmospheric Research, Boulder, United States

Corresponding author: suvarna@tropmet.res.in

Supplementary Tables and Figures

Table-S1: List of moderate-large-volcanic eruptions (VEI ≥3) that occurred in the tropical region (30ºS-30ºN) during 1870-2017 (https://volcano.si.edu/)

Sr No.	volcano	year	Location	(VEI)	Rainfall condition	El Niño/La Niña
1.	Cotopaxi	June 1877	0.68 ºS, 87.44 ºW	4	drought in the current yr.	El Niño in the current yr.
2.	Suwanosejima	1877	29.38 ºN 129.43 ºE	4	drought in the current yr.	El Niño in the current yr.
3.	De Fuego	June 1880	14.46 ºN, 90.88 ºW	4	deficit in the current yr.	-
4.	Krakatoa	August 1883	6.10 ºS, 105.42 ºE	6	wet followed by deficit.	-
5.	Tungurahua	January 1886	1.74 ºS, 78.44 ºW	5	wet followed by deficit.	El Niño within 2 yrs.
6.	Mount Tarawera	June 1886	15.60 ºS, 175.06 ºW	4	wet followed by deficit	El Niño with 2 yrs.
7.	Colima	August 1889	19.51 ºN, 103.62 ºE	4	wet followed by deficit.	La Niña in current yr. and El Niño after 2 yrs.
8.	Suwanosejima	October 1889	29.38 ºN, 129.43 ºE	4	wet followed by deficit	La Niña in the current yr. and El Niño after 2 yrs.

9.	Mount Wurlali	June 1892	7.7ºS, 128.40 ºE		Flood for 2 yrs.	La Niña in the current yr and after 1 yrs
10.	Ruiz	1895	4.57° N, 74.29° W	3	deficit condition for 2 yrs.	El Niño in next yr
11.	Mt Mayon	June 1897	13.26ºN, 113.69 ºW		Wet for 2 yrs.	
12.	Dona Juana	November 1899	1.47 °N, 76.92 °W	4	Wet followed by drought.	El Niño in the current yr. and next yr.
13.	Mount Pelée	May 1902	14.82 ºN, 61.17ºW	4	drought within 2 yrs.	El Niño in current yr.
14.	La Soufrière(Saint Vincent)	May 1902	13.2 ºN, 16.1ºW	4	drought in 2 yrs.	El Niño in current yr.
15.	Lolobau	August 1904	4.92ºS, 151.16 ºE	4	drought for 2 yrs.	El Niño in current yr.
16.	Lolobau	1911	4.92 ºS, 151.16 ºE	4	drought in current yr.	El Niño in the current yr.
17.	Tungurahua	March 1916	1.47 ºS 78.4ºW	6	flood for 2 yrs.	La Niña in the current yr.
18.	Agrihan	April 1917	18.77 ºN, 145.67ºE	4	flood followed by drought.	El Niño in next yr.
19.	Kelud	May 1919	7.5 ºS, 112.18 ºE	4	wet followed by drought.	-
20.	Manam	August 1919	4.08 ºS, 145.04ºE	4	wet followed by drought.	-
21.	Tandikat	April 1924	0.433ºS, 100.317 ºE		wet followed by deficit	El Niño in next yr.
22.	Irmote-jima	October 1924	24.56 ºN ,124.00 ºE	4	wet followed by deficit	El Niño in next yr.
23.	Volcan De Fuego	January 1932	14.46 ºN, 90.88 ºW	4	deficit followed by wet.	-
24.	Suoh	July 1933	5.25 ºS, 104.27ºE	4	flood in the current yr.	La Niña in current yr. and El Niño within 2 yrs.
25.	Ambrym	December 1950	16.25ºS, 168.12ºE	4+	wet followed by drought.	El Niño in the next yr.
26.	Lamington	January 1951	8.5 7ºS, 148.09ºE	4	drought in current yr.	El Niño in current yr.
27.	Kelud	August 1951	7. 93 ºS, 112.31 ºE	4	drought in current yr.	El Niño in current yr.
28.	Bagana	February 1952	6.10 ºS, 152.20 ºE	4	deficit in current yr.	La Niña after 2 yrs.
29.	Mount Agung	May 1963	8.34 ºS, 115.5ºE	5	Wet for 2 yrs.	
30.	Taal	September 1965	14.01 ºN, 120.99 ºE	4	drought in current yr.	El Niño in current yr.
31.	Kelud	April 1966	7.5ºS, 112.18 ºE	4	drought in current yr.	El Niño with 2 yrs.
32.	Mount Awu	August 1966	3.67 ºS, 125.50 ºE	4	drought in current yr.	El Niño with 2 yrs.
33.	Fernandina Island	June 1968	0.37 ºS, 91.55ºW	4	drought in current yr.	El Niño in current yr.

34.	Volcan De Fuego	October 1974	14.46 ºN, 90.88 ºW	4	drought in current yr.	La Niña in the next yr. and El Niño within 2 yrs.
35.	Alaid	April 1981	14.88º N, 39.92 ºW	4	deficit followed by drought	El Niño in the next yr.
36.	Mount Pagan	May 1981	18.13N 145.8E	4	deficit followed by drought.	El Niño in the next yr.
37.	El-chichon	March1982	17.35 ºN, 93.23 ºW	5	drought in the current yr.	El Niño in the current yr.
38.	Galunggung	May 1982	7.25 ºS 108.06ºE	4	drought in the current yr.	El Niño in the current yr.
39.	Colo	July 1983	0.17ºS, 121.61ºE	4	wet followed by deficit	La Niña in the current yr.
40.	Nevado del Ruiz	November 1985	4.53ºN, 75.19ºW	3	drought for 3 yrs.	El Niño within 2 yrs.
41.	Kilauea	June 1987	19.4 ºN, 155.2ºW		drought in current yr.	El Niño in the current yr and La Niña in the next yr.
42.	Kelud	February 1990	7.93 ºS, 112.31ºE	4	wet followed by deficit	El Niño in the next yr.
43.	Mt Pinatubo	June 1991	15.13 ºN, 120.35 ºW	6	deficit for 2 yrs.	El Niño in current yr.
44.	Lascar	April 993	23.37ºS, 67.73ºW	4	wet for 2 yrs.	-
45.	Rabaul	September 1994	4.27 ºS, 152.20 ºE	4	flood followed by deficit	
46.	Soufriere Hills	June 1997	16.72ºN, 62.18 ºE	4	wet followed by deficit	El Niño in the current yr.
47.	Reventador	November 2002	0.08 ºS,77.66 ºE	4	drought in the current yr.	El Niño in the current yr.
48.	Ruang	September 2002	2.30 ºN, 125.37ºE	4	drought in the current yr.	El Niño in the current yr.
49.	Manam	November 2004	4.08 ºS, 145.04 ºE	4	drought in the current yr.	El Niño within 2 yrs.
50.	Rabaul	October 2006	4.27 ºS, 152.20 ºE	4	wet for 2 yrs.	El Niño in the current yr.
51.	Merapi	November 2010	7.54ºS, 110.44 ºE	4	wet followed by deficit	La Niña for two consecutive yrs.
52.	Nabro	June 2011	13.37 ºN, 41.70 ºE	4	wet followed by deficit	La Niña in the current yr.
53.	Kelud	February 2014	7. 93ºS, 112.31ºE	4	drought for 2 yrs.	El Niño in the next yr.

Table-S2: List of moderate-large volcanic eruptions (VEI ≥3) that occurred in the Northern and Southern hemisphere extra-tropics (30-90ºN-S) during 1870-2017 (https://volcano.si.edu/).

Sr. No.	Volcano	Month year	Location	Volcanic Explosivity Index (VEI)	Rainfall Condition	El Niño/La Niña
1.	Sinarka	1872 (not sure but shown as January in plot)	48.89 ºN, 104.30º W	4	Wet in the current yr.	
2.	Grímsvötn	1873 (month of eruption is not sure but shown as January in plot)	64.52 ºN, 17.33 ºW	4	Drought in the current yr.	
3.	Askja	March 1875	65.03ºN, 16.75ºW	5	Wet in the current yr.	
4.	Mt. Augustine	October 1883	59.36ºN, 153.43 ºW	4	Wet in the current yr.	
5.	Mount Tarawera	June 1886	38.13ºS, 176.00 ºE	4	Wet in the current yr.	
6.	Bandai	July 1888	37.60 ºN, 140.07 ºE	4	deficit in the current yr.	El Niño in the current yr.
7.	Calbuco	January 1893	41.33 ºS, 72.61ºW	4	Wet in the current yr.	La Niña within two yrs.
8.	Grímsvötn	May 1903	64.92 ºN 17.33ºW	4	Wet in the current yr.	El Niño within two yrs.
9.	Ksudach	March 1907	50.80 ºN, 157.53 ºE	5	deficit in the current yr followed by wet	La Niña for Next three yr
10.	Novarupta	June 1912	58.16 ºN, 155.92 ºW	6	deficit in the current yr.	El Niño within two yrs.
11.	Sakurajima	January 1914	31.59 ºN, 130.66 ºW	4	Wet in the current yr.	
12.	Katla	October 1918	63.38ºN, 19.03 ºW	4+	wet in the 1919 yr.	El Niño in 1918.
13.	Raikoke	February 1924	48.29 ºN, 153.25 ºE	4	Wet in the current yr.	El Niño within two yrs.
14.	Avachinsky	April 1926	53.26 ºN, 158.83 ºE	4	Wet in current yr.	
15.	Komagatake	June 1929	42.06 ºN, 140.68 ºE	4	deficit in the current yr.	El Niño within two yrs.
16.	Kliuchevskoi	March 1931,	56.06 ºN, 160.64 ºE	4	Wet in the current yr.	
17.	Aniakchak	May 1931	56.88 ºN, 158.17 ºE	4	Wet in the current yr.	
18.	Cerroo Azul	1932	35.65 ºS, 70.76 ºW	5+	deficit in the current yr.	La Niña within two yrs.
19.	Kuchinoerabujima	December 1933	30.44 ºN, 130.22 ºE	4	Wet in 1934.	La Niña in 1933.
20.	Kharimkotan	1933	49.12 ºN, 154.50 ºE	5	Flood in the current yr.	La Niña in the current yr.

21.	Avachinsky	February 1945	56.88 ⁰N, 158.17 ⁰E	4	Wet in the current yr.	
22.	Sarychev Peak	November 1946	48.09 ⁰N, 153.20 ⁰E	4	Flood in 1947.	
23.	Hekla	March 1947	63.98 ⁰N, 19.70 ⁰W	4	Flood in current yr.	
24.	Mt Spurr	July 1953	61.30 ⁰N, 152.25 ⁰W	4	Wet in the current yr.	
25.	Carran-Los Venados	July 1955	40.30 ⁰S, 72.07 ⁰W	4	Flood in current yr.	La Niña within two yrs.
26.	Bezymianny	March 1956	55.58 ⁰N, 160.35 ⁰E	5	Flood in the current yr.	La Niña in current yr. and within two yrs.
27.	Shiveluch	November 1964	56.65 ⁰N, 161.36 ⁰E	4+	Drought in 1965 yrs.	
28.	Tyatya	July 1973	44.21 ⁰N, 146.15 ⁰E	4	Wet in the current yr.	La Niña in the current yr.
29.	Tolbachik	July 1975	55.49 ⁰N, 160.19 ⁰E	4	Flood in in the current yr.	La Niña in the current yr.
30.	Mt Augustine	January 1976	59.36 ⁰N, 153.43 ⁰W	4	Deficit condition in the current yr.	El Niño in the current yr.
31.	Mt Helens	May 1980	46.19 ⁰N, 122.19 ⁰W	5	Wet in the current yr.	
32.	Mt Augustine	March 1986	59.36 ⁰N, 153.43 ⁰W	4	Drought in current yr.	El Niño in next yr.
33.	Chikurachki	November 1986	50.33 ⁰N, 155.46E ⁰W	4	Drought in 1987.	El Niño in next yr.
34.	Kliuchevskoi	February 1987	56.06 ⁰N, 160.64 ⁰E	4	Drought in the current yr.	El Niño in the current yr.
35.	Mt Hudson	August 1991	74.33 ⁰S, 99.42 ⁰W	5+	Deficit in current yr.	El Niño in current yr.
36.	Mt. Spurr	June 1992	61.30 ⁰N, 152.25 ⁰W	4	deficit in the current yr.	
37.	Shiveluch	June 2001	56.65 ⁰N,161.36⁰ W	4	deficit in the current yr.	El Niño within two yrs.
38.	Mt okmok	July 2008	53.48 ⁰N, 168.17 ⁰W	4	Wet in the current yrs.	El Niño within two yrs.
39.	Chaiten	May 2008	42.83 ⁰S, 72.65 ⁰W	4	Wet in the current yr.	El Niño within two yrs.
40.	Kasatochi	August 2008	55.00 ⁰N, 175.00 ⁰E	4	Wet condition in the current yr.	El Niño within two yrs.
41.	Sarychev	June 2009	48.00 ⁰N, 153.20 ⁰W	4	Drought in the current yr.	El Niño in the current yr.
42.	Eyjafjallajokull	April 2010	63.63 ⁰N, 19.60 ⁰W	4	Wet condition in current yr.	La Niña In current yr.

43.	Grimsvotn	May 2011	64.42 ^{o}N,<br>17.33 ^{o}W	4	Wet in the current yr.	La Niña in current yr.
44.	Puyehue-Cordon Caulle	June 2011	40.59 ^{o}S,<br>72.12 ^{o}W	5	Wet in the current yr.	La Niña in the current yr.
45.	Etna	April(March) 2013	37.89 ^{o}N, 137.48 ^{o}E	3	Flood in current yr.	
46.	Mt Ontake	September 2014	35.89 ^{o}N, 137.48 ^{o}E	3	Drought in the current yr.	El Niño within two yrs.
47.	Calbuco	April 2015	41.33 ^{o}S, 72.60 ^{o}W	4	drought in the current yr.	El Niño in the current yr.

Table-S3 Description model experiments

Sr No.	Name of the Experiment	Sea surface temperature (sst)	Period of simulations	Volcanic aerosols injected
1	ECHAM6-HAMMOZ Vol simulations	SST Monthly varying SST during January 2010 to December 2013	Ten members ensemble mean for 1 January 2010 to 10 January 2010. Simulations ends on 31 December 2013	At the model levels between 10 to 15 km
2	ECHAM6-HAMMOZ CTL simulations	SST Monthly varying SST during January 2010 to December 2013	Ten members ensemble mean for 1 January 2010 to 10 January 2010. Simulations ends on 31 December 2013	No Volcanic aerosols
3	The MPI-ESM model simulations (Vol)	Simulated by MPI-ESM	Ten members Ensemble mean for 1 January 2010 to 10 January 2010. Simulations ends on 31 December 2013	Volcanic aerosol radiative properties from ECHAM6-HAMMOZ Vol simulations
4	The MPI-ESM model simulations (CTL)	Simulated by MPI-ESM	Ten members ensemble mean for 1 January 2011 to 10 January 2011. Simulations ends on 31 December 2013	Volcanic aerosol radiative properties from ECHAM6-HAMMOZ CTL simulations

Supplementary Figures

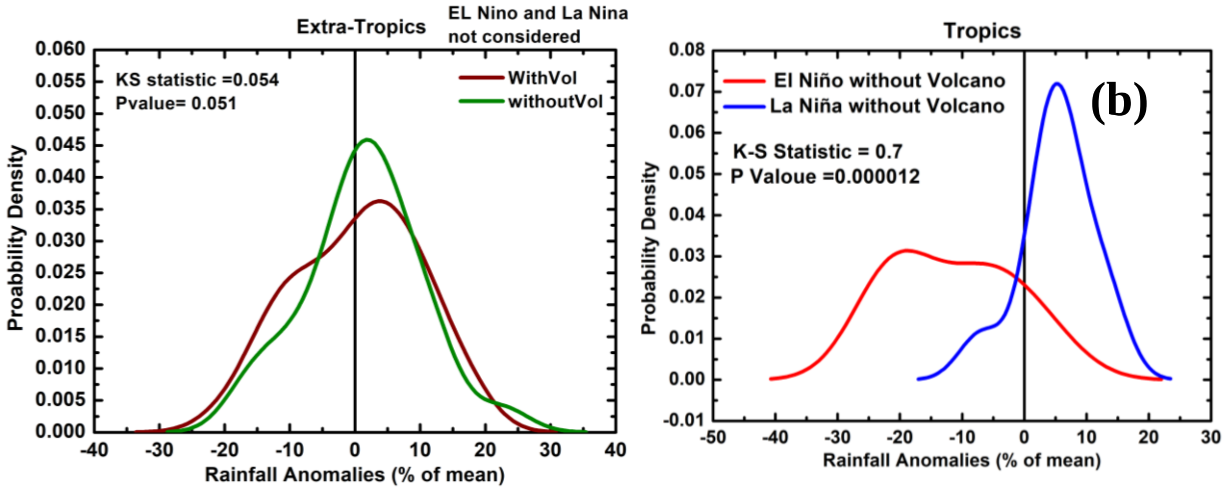

Figure S1: Probability distribution of rainfall anomalies (1871 – 2016) (a) stratified with and without volcanic eruptions within two years of eruption in the extra tropics when El Niño and La Niña years are not considered, (b) when stratified without volcanic eruptions in the tropics for El Niño and La Niña years. The statistical measures of Kolmogorov-Smirnov (K-S) test shown in Fig.a-b indicate that distributions are distinct (Figure created using the COLA/GrADS software).

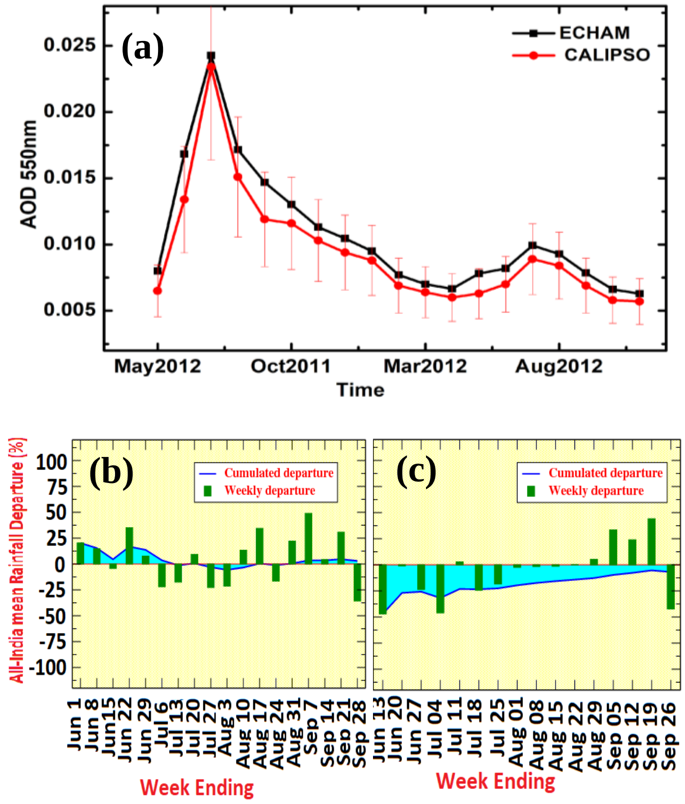

Figure S2: (a) Time series of anomalies in stratospheric AOD at 550 nm (>350K) from Vol simulation and CALIPSO measurements. Weekly departure of rainfall in India Meteorology Department (IMD) data for the monsoon in (b) 2011, and (c) 2012 (Figure created using the COLA/GrADS software).

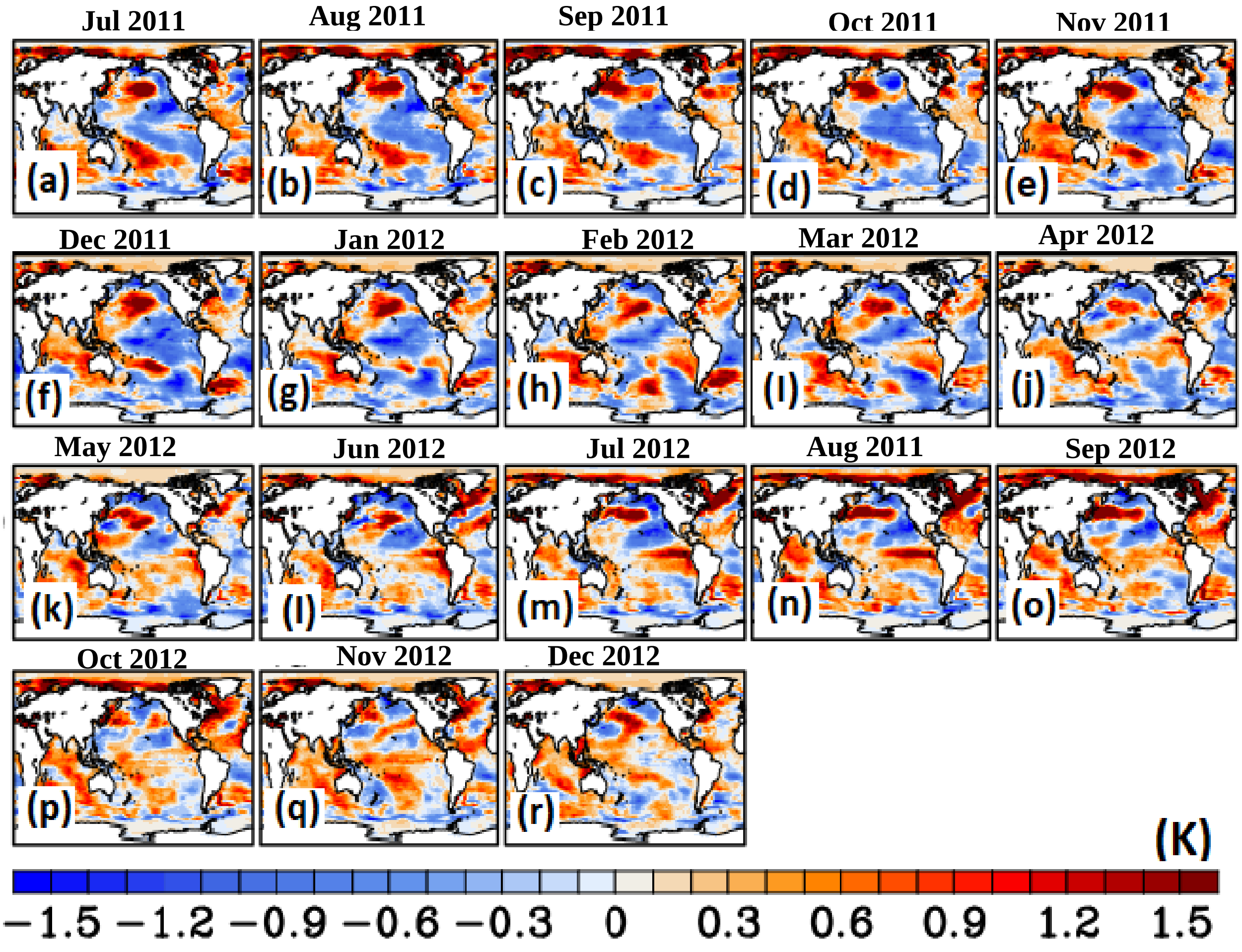

Figure S3: Distribution of monthly mean anomalies in sea-surface temperature (K) for (a) – (r) July 2011 to December 2012 from Hadisst data (climatology used for the period 1980 – 2016) (Figure created using the COLA/GrADS software).

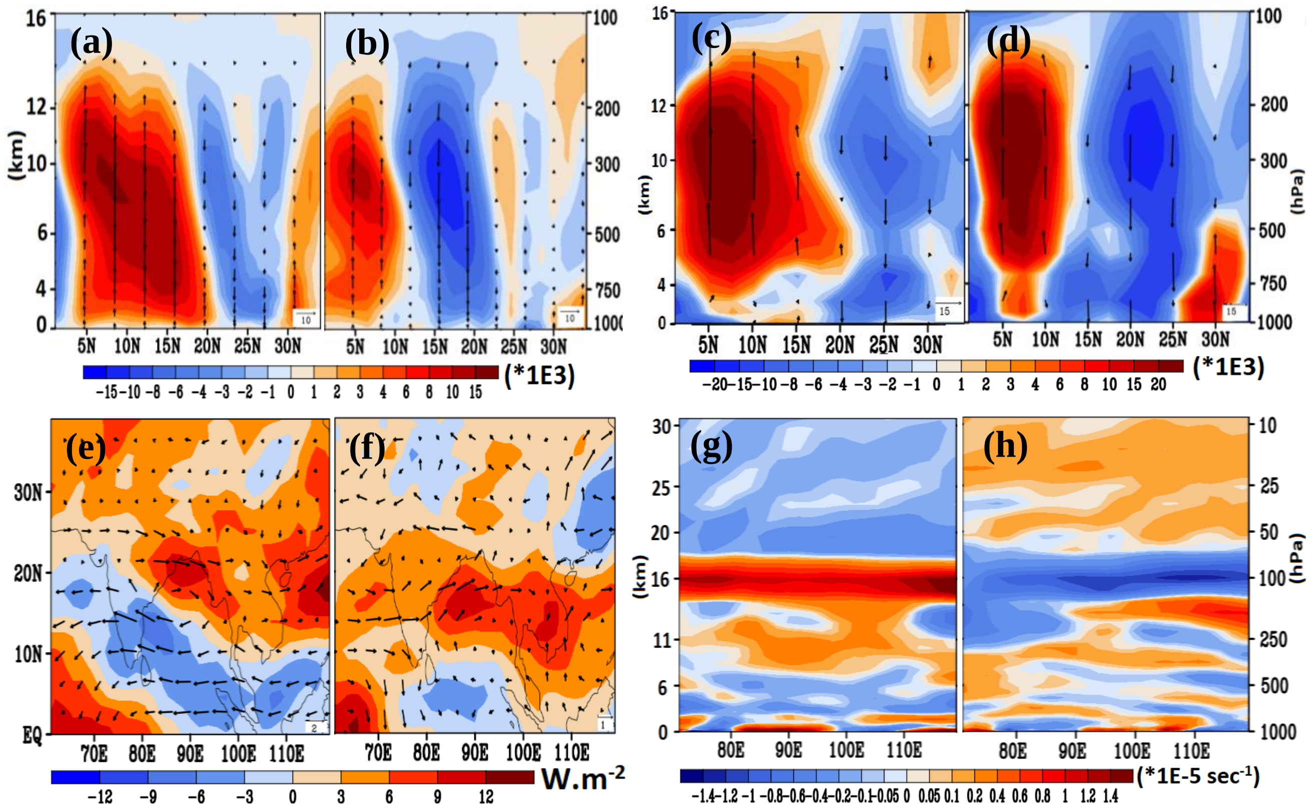

Figure S4: Latitude–pressure section of anomalies from ECHAM6-HAMMOZ simulations (Vol-CTL) of vertical velocity (averaged for 70 – 100 ºE, vertical (velocities are scaled by 1000) from ECHAM6-HAMMOZ average for monsoon (a) 2011, (b) 2012 (winds are shown by vectors). (c)-(d) same as (a)-(b) but from NCEP data. Anomalies of Outgoing Long-wave Radiations (OLR) ($W.m^{-2}$) from ECHAM6-HAMMOZ simulations (Vol-CTL) averaged for monsoon (e) 2011, (f) 2012, and winds at 850 hPa indicating low level monsoon jet. Longitude–pressure section for anomalies of Brunt Väisäla frequency (1E-5 sec^{-1}) from ECHAM6-HAMMOZ simulations (Vol-CTL) averaged for (20 – 35 ºN) and for monsoon season (g) 2011, (h) 2012. (Figure created using the COLA/GrADS software).

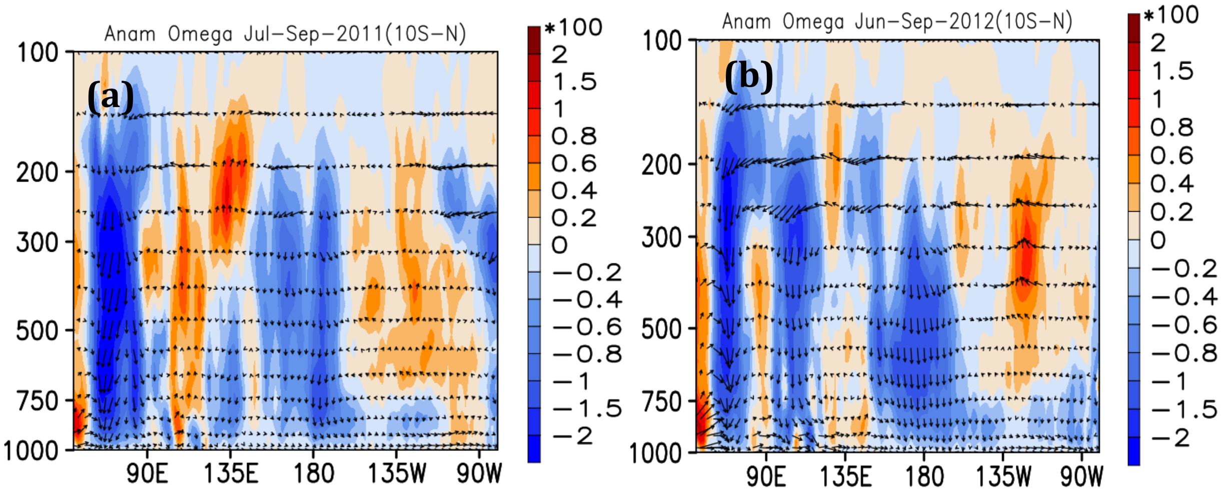

Figure S5: Longitude–pressure section of anomalies of vertical velocity (averaged for 10ºS – 10 ºN, vertical (velocities are scaled by 100) from ECHAM6-HAMMOZ (Vol-CTL) average for (a) July-September 2011, (b) June-September 2012 (winds are shown by vectors) (Figure created using the COLA/GrADS software).

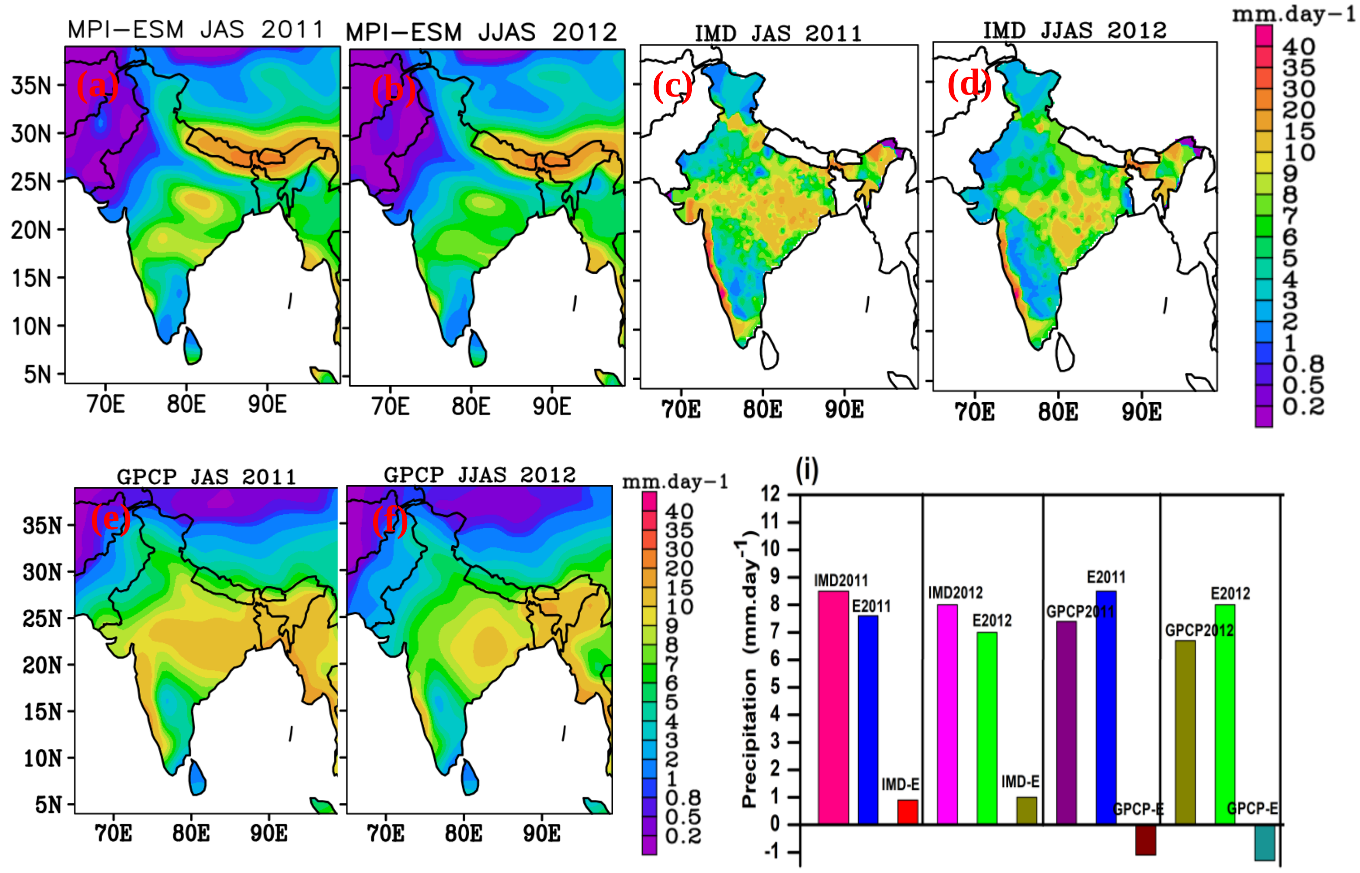

Figure S6: Distribution of precipitation (mm.day^{-1}) from MPI-ESM (Vol) simulations averaged for (a) July-September 2011, (b) June-September 2012, (c)-(d) same as (a)-(b) but from IMD rain gauge measurements, (e)-(f) same as (a)-(b) but from GPCP. (i) Comparison of precipitation from MPI-ESM Vol simulation with IMD and GPCP data averaged for July-September 2011 and June-September 2012 and Indian region (78 – 93 ^{o}E, 8 – 35 ^{o}N), “E2011 and E2012” indicates MPI-ESM Vol simulation. Departure of simulated rainfall from IMD (IMD-E), and GPCP (GPCP-E) is shown as a last bar in each section (Figure created using the COLA/GrADS software).

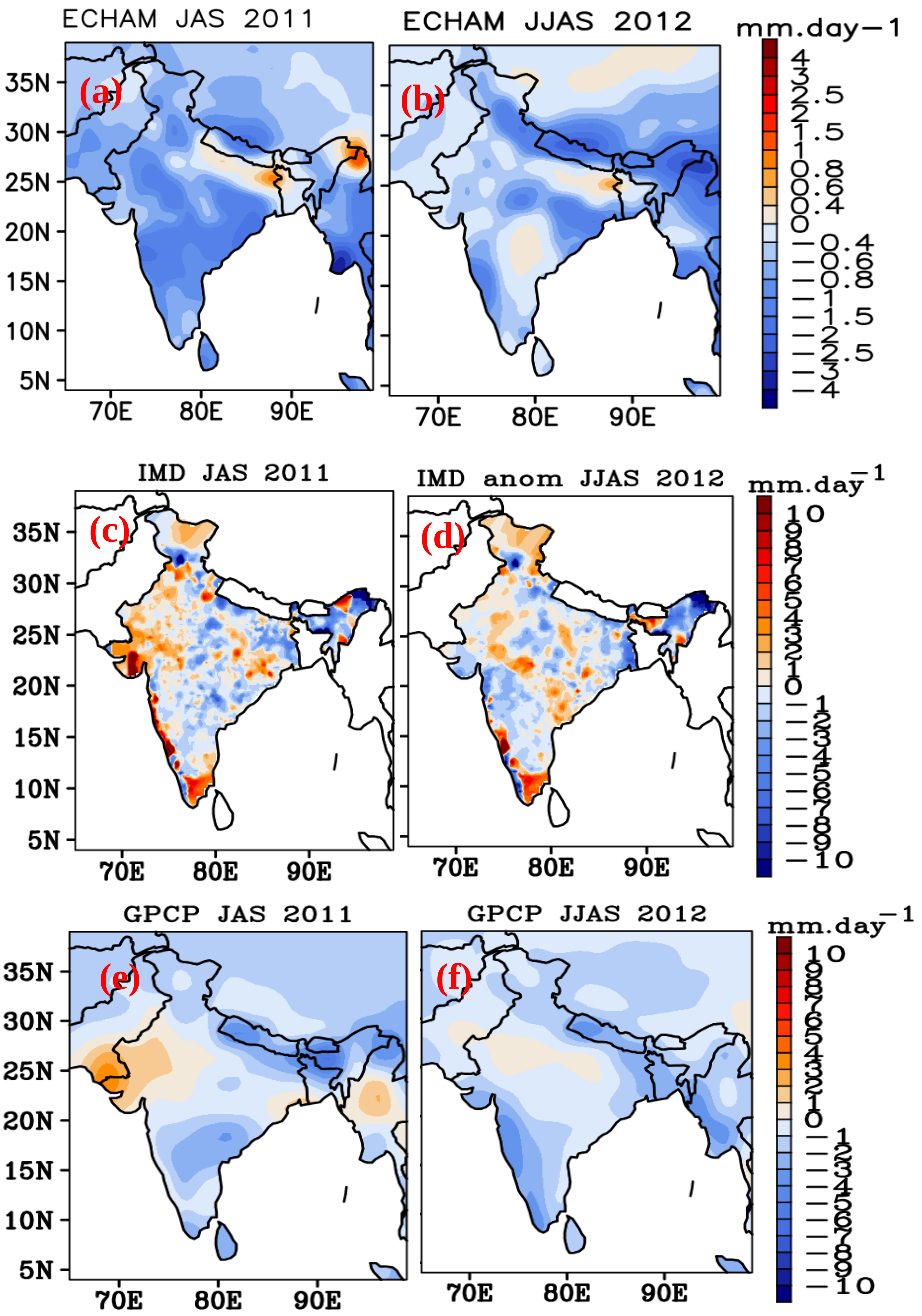

Figure S7: Distribution of anomalies of rainfall (mm.day^{-1}) averaged for July-September 2011, and June-September 2012 from (a)-(b) MPI-ESM (Vol-CTL); (c)-(d) IMD (climatology used for years 1950 – 2015), (e)-(f) GPCP (climatology used for 1981 – 2015) (Figure created using the COLA/GrADS software).

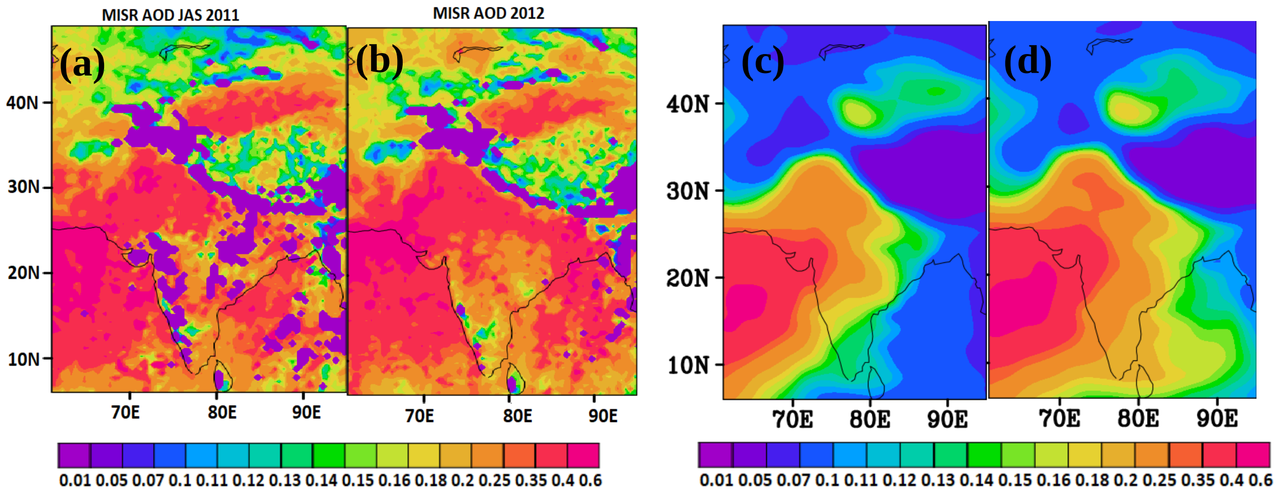

Figure S8: Distribution of aerosol optical depth (AOD) from MISR satellite averaged for monsoon and (a) July-September 2011, (b) June-September 2012. (c) – (d) same as (a) – (b) but from ECHAM6-HAMMOZ Vol simulation (Figure created using the COLA/GrADS software).

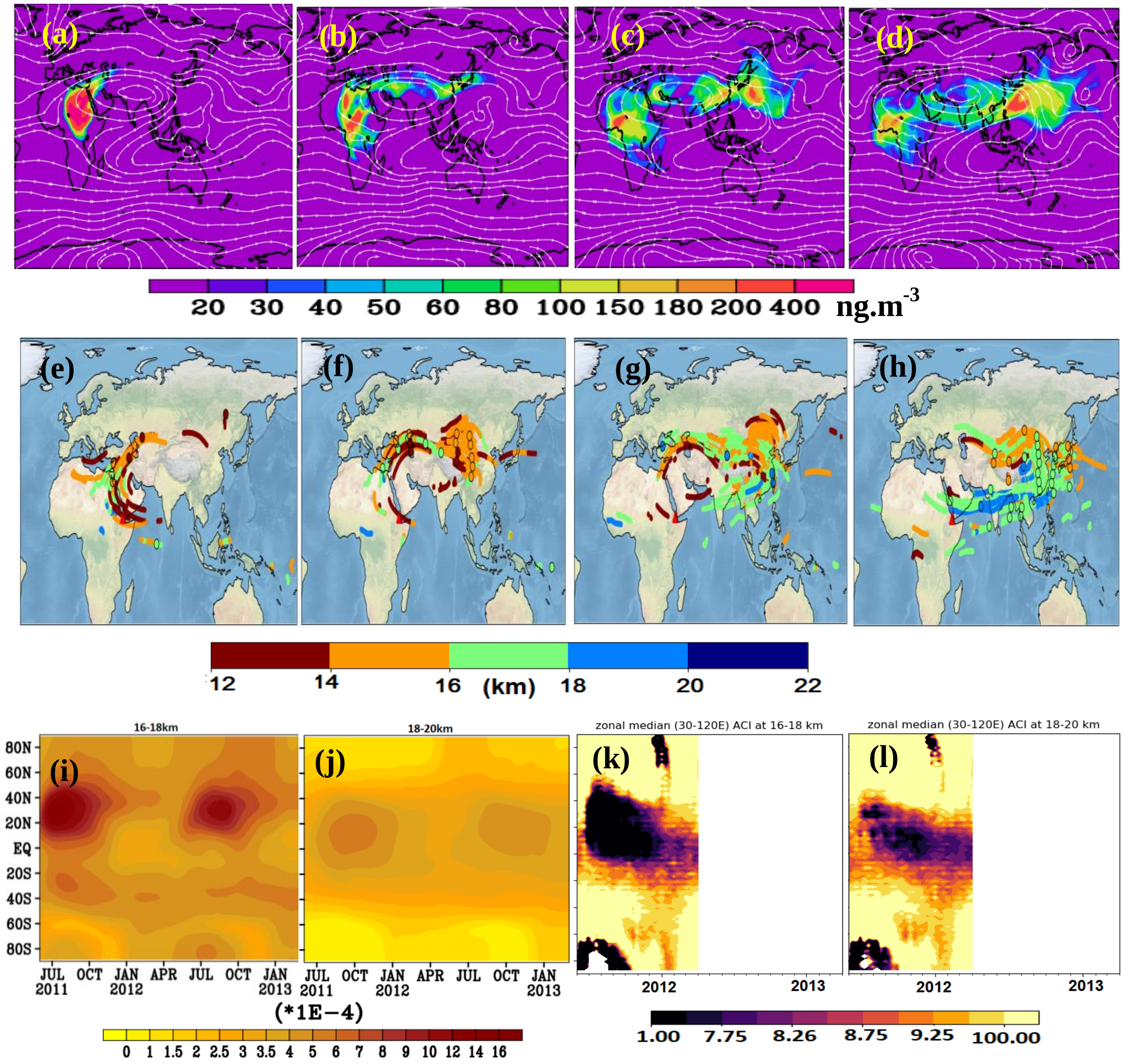

Figure S9: Anomalies of sulfate aerosols (ng.m-3) from the ECHAM6-HAMMOZ simulations (Vol - CTL) averaged for 12 – 20 km on (a) 14 June, (b) 16 June, (c) 18 June, and (d) 20 June 2011. (e)-(h) MIPAS sulfate aerosol observations (black circles) on the same days as (a)-(d) and MIPAS observations within ±3days mapped to each day using Lagrangian forward and backward trajectories (coloured circles). Distribution of aerosol extinction from the ECHAM6-HAMMOZ Vol simulations averaged over the Asian summer monsoon region 30 – 120 ºE and (i) 16 – 18 km, (j) 18-20 km. Distribution of ACI index from MIPAS satellite measurements during January 2011 to March 2012 averaged over the Asian summer monsoon region 30 – 120 ºE and between (k) 16 – 18 km, (l) 18 – 20 km. (Figure created using the COLA/GrADS software).
